# High-resolution genotyping and mapping of recombination and gene conversion in the protozoan *Theileria parva* using whole genome sequencing

**DOI:** 10.1186/1471-2164-13-503

**Published:** 2012-09-23

**Authors:** Sonal Henson, Richard P Bishop, Subhash Morzaria, Paul R Spooner, Roger Pelle, Lucy Poveda, Martin Ebeling, Erich Küng, Ulrich Certa, Claudia A Daubenberger, Weihong Qi

**Affiliations:** 1 International Livestock Research Institute, Nairobi, 00100, Kenya; 2 Functional Genomics Center Zurich, UZH/ETHZ, Winterthurerstrasse 190, 8057, Zurich, Switzerland; 3 F. Hoffmann-La Roche AG, Basel, 4070, Switzerland; 4 Swiss Tropical and Public Health Institute, Socinstrasse 57, Basel, 4002, Switzerland; 5 University of Basel, Peterplatz 1, Basel, 4003, Switzerland

## Abstract

**Background:**

*Theileria parva* is a tick-borne protozoan parasite, which causes East Coast Fever, a disease of cattle in sub-Saharan Africa. Like *Plasmodium falciparum*, the parasite undergoes a transient diploid life-cycle stage in the gut of the arthropod vector, which involves an obligate sexual cycle. As assessed using low-resolution VNTR markers, the crossover (CO) rate in *T. parva* is relatively high and has been reported to vary across different regions of the genome; non-crossovers (NCOs) and CO-associated gene conversions have not yet been characterised due to the lack of informative markers. To examine all recombination events at high marker resolution, we sequenced the haploid genomes of two parental strains, and two recombinant clones derived from ticks fed on cattle that had been simultaneously co-infected with two different parasite isolates.

**Results:**

By comparing the genome sequences, we were able to genotype over 64 thousand SNP markers with an average spacing of 127 bp in the two progeny clones. Previously unrecognized COs in sub-telomeric regions were detected. About 50% of CO breakpoints were accompanied by gene conversion events. Such a high fraction of COs accompanied by gene conversions demonstrated the contributions of meiotic recombination to the diversity and evolutionary success of *T. parva*, as the process not only redistributed existing genetic variations, but also altered allelic frequencies. Compared to COs, NCOs were more frequently observed and more uniformly distributed across the genome. In both progeny clones, genomic regions with more SNP markers had a reduced frequency of COs or NCOs, suggesting that the sequence divergence between the parental strains was high enough to adversely affect recombination frequencies. Intra-species polymorphism analysis identified 81 loci as likely to be under selection in the sequenced genomes.

**Conclusions:**

Using whole genome sequencing of two recombinant clones and their parents, we generated maps of COs, NCOs, and CO-associated gene conversion events for *T. parva*. The data comprises one of the highest-resolution genome-wide analyses of the multiple outcomes of meiotic recombination for this pathogen. The study also demonstrates the usefulness of high throughput sequencing typing for detailed analysis of recombination in organisms in which conventional genetic analysis is technically difficult.

## Background

The tick-transmitted protozoan *Theileria parva* is an apicomplexan parasite that is phylogenetically distantly related to the human malaria parasite *Plasmodium falciparum*[1]. *T. parva* causes East Coast fever (ECF), a costly tick-borne disease that kills about 1 million cattle per year in eastern, central and southern Africa [1]. ECF threatens some 25 million cattle in 11 countries and is now putting at risk a further 10 million animals in new regions, such as southern Sudan, where the parasite has recently been discovered to be endemic [2].

The life cycle of *T. parva* involves transmission of the sporozoite stage to cattle or buffalo in the saliva of feeding adult and nymphal *Rhipicephalus appendiculatus* ticks [3]. Within the bovidae host, sporozoites invade lymphocytes and this ensuing multi-nucleate schizont stage immortalizes infected lymphocytes and divides in synchrony with them, ensuring that the parasite is transmitted to each daughter cell. A proportion of schizonts develop subsequently into merozoites followed by the piroplasm stage that is infective to the *R. appendiculatus* ticks. After ingestion by the tick, free piroplasms develop into micro- and macro-gametes that undergo syngamy to form the diploid zygotes [4,5]. Zygotes undergo meiotic division accompanied by sexual recombination resulting in the development of the kinete stage that later invades tick salivary gland type III acinar cells. Sporozoites differentiate within these acini and become the infective stage again for mammalian hosts [3].

Vaccination against ECF is achieved by the live “infection and treatment” immunisation method that induces potent cytotoxic T cell responses against the schizont stage of the parasite [6]. This immune protection is partially *T. parva* isolate-specific, dominated by responses targeting a limited range of antigens in the vaccinated animal and vulnerable to breakthrough infection upon challenge [7,8]. For obligate sexually reproducing eukaryotic pathogens with a complex life cycle in arthropod vectors and mammalian hosts, a map of recombination events is essential for understanding parasite genome evolution. Such data provide insight into the mechanisms of immune evasion and host and parasite co-evolution. In-depth studies for *T. parva* are at an early stage due to lack of informative high-resolution markers. The approaches previously used to dissect *T. parva* parasite polymorphism and recombination events include monoclonal antibodies [9], gene probes binding to multi-copy loci [5], panels of micro- and mini-satellite markers [10,11] and most recently the sequence polymorphism in antigens that are targets of CD8+ T cell responses [12]. While useful to generate preliminary information, these techniques were unable to provide high resolution dissection of recombination events at the whole genome level.

Recent experimental analyses performed in *T. parva* based on a panel of Variable Number Tandem Repeat (VNTR) and PCR-Restriction Fragment Length Polymorphism (RFLP) markers [10,13] indicate high frequencies of crossover events. An initial linkage map of *T. parva* has been constructed using 79 VNTR markers and 35 recombinant clones derived from ticks fed on cattle co-infected with Muguga and Marikebuni stocks [10]. The average genome-wide crossover rate was calculated to be approximately three times higher than that in *Plasmodium falciparum*. In total 434 crossover (CO) events, ten recombination hot spots and 13 cold spots were identified [10].

Massively parallel DNA sequencing technologies are becoming affordable for genotyping and have been applied to map recombination events by sequencing progeny strains and their parents across a wide range of different taxa. These include humans [14,15], rice [16], yeast [17,18], *Toxoplasma gondii*[19] and most recently *P. falciparum*[20]*.* The elucidation of the *T. parva* (Muguga) genome sequence [1] provides a reference template for the generation of a genome-wide panel of single nucleotide polymorphism (SNP) markers for high-resolution genotyping.

We describe herein the whole genome sequencing of two recombinant clones resulting from co-infection experiments of cattle with different *T. parva* isolates. With the panel of high density SNP markers established via sequencing, we identified crossover and non-crossover events, as well as CO-associated gene conversions. Intra-species genome comparison identified a preliminary list of genes undergoing positive selection, which can be useful for the future systematic analysis of *T. parva* genes involved in co-evolution with its tick vector and its cattle and cape buffalo (*Syncerus caffer*) hosts.

## Results and discussion

### Whole genome sequencing, mapping and *de* novo assembly

**Figure 1:**
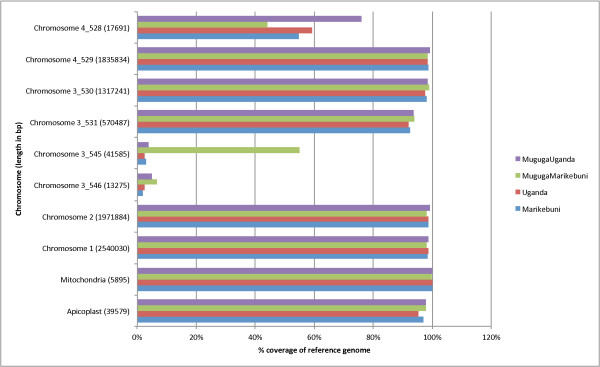
**Coverage of *T. parva *Muguga reference genome by 454 reads in sequenced strains.** X axis shows the percentage of reference coverage by 454 reads. Y axis represents the chromosomes. The numbers in parenthesis next to chromosome names are genomic sizes in base pairs. In the current *T. parva* genome assembly, chromosome 3 consists of four super contigs, chromosome 4 consists of two supercontigs, as shown by the y axis of the figure.

In this study, we sequenced four *T. parva* isolates using the Roche 454 GS FLX Titanium chemistry (Table 1), which included two parental parasite isolates, Marikebuni and Uganda, and two recombinant clones derived from experimental co-infection experiments of cattle. After tick pick up, sporozoites were isolated from tick salivary glands and used to re-infect *T. parva*-naive cattle to finally recover purified piroplasms for DNA sequencing. The two recombinant clones sequenced here were denoted MugugaMarikebuni and MugugaUganda. The other parent for both recombinant clones was *T. parva* Muguga, the reference isolate previously sequenced using the Sanger shotgun method [1]. The sequencing coverage ranged from 16 to 59 fold, with an average of 45 fold. The 454 reads were first mapped to the Muguga genome and a mapped assembly was obtained for each strain (Table 2). Although more than 98% of the Muguga genome could be mapped with 454 reads for each of the strains, the sub-telomeric regions of chromosomes 3 and 4 (contigs chromosome3_545, chromosme3_546, chromosome4_528) were less well covered (Figure 1, Additional file 1: Figure S5), as were the sub-telomeric regions of chromosome 1 (i.e. the loci of *Sfi*I ST-c1a and Subtelomeric Variable Secreted Protein (SVSP)-c1a, data not shown). These regions contain hyper-variable multi-copy gene families, such as the tandemly arrayed Tpr family on chromosome 3, which could only be partially assembled in the reference genome [1], and sub-telomeric gene families, which are known to be isolate-specific in some instances [21]. Since the mapping algorithm randomly assigned repetitive reads to one of the mapped regions, unmapped regions were most likely due to highly variable sequences in the strains, although some might also be due to low complexity sequences (Additional file 2: Table S3).

**Table 1:** **Sequencing of *T. parva *parent and progeny strains using the Roche 454 GS FLX sequencer with Titanium chemistry**

**Strain**	**Library type**	**Number of reads**	**Number of bases**	**Mean length (nt)**	**Median length (nt)**	**Mode length (nt)**	**Average coverage**
Marikebuni	Shotgun	1164490	424965409	365	394	268	59
	PE (8 Kb) ^1^	473070	148973321	315	321	336	
Uganda	Shotgun	740576	304223344	411	440	464	46
	PE (8 Kb)	272148	90236805	332	340	358	
MugugaMarikebuni	Shotgun	237978	88212143	371	395	417	16
	PE (11 Kb)	433252	115642514	267	269	263	
MugugaUganda	Shotgun	732222	277159312	379	397	410	57
	PE (3 Kb)	608039	213454970	351	368	389	

^1^Paired-end library (insert size in Kb).

**Table 2:** **Comparison of reference assembly and *de novo *assembly of *T. parva *parent and progeny genomes**

**Strain**	**Assembly**	**Number of scaffolds**	**N50 scaffolds (nt)**	**Number of contigs (nt)**^**1**^	**N50 contigs (nt)**^**1**^	**Assembled bases (nt)**^**1**^
Marikebuni	Reference	-	-	399	90892	8067780
	*De novo*	31	1841172	339	175119	8457331
Uganda	Reference	-	-	416	104202	8072088
	*De novo*	26	2000289	268	153816	8326153
MugugaMarikebuni	Reference	-	-	447	88526	8074124
	*De novo*	50	1846792	396	56601	8195173
MugugaUganda	Reference	-	-	288	165046	8138418
	*De novo*^2^	30	1209478	232	235334	8333992

^1^Only contigs with length above 100 nt were included.

^2^ Assembled with 454 gsAssembler version 2.5.3.

The 454 reads were also assembled *de novo* into scaffolds and contigs (Table 2). For all isolates except MugugaMarikebuni, the N50 contig length produced by the *de novo* assembly was higher compared to that of mapped assembly, indicating that *de novo* assembly was less fragmented. The total numbers of bases assembled were also significantly higher (P-value < 0.05) in all isolates for the *de novo* assembly compared to those in the mapped assemblies, irrespective of the difference in sequence coverage between isolates. This also suggested the presence of highly divergent sequences among the strains, from which the reads could not be mapped back to the reference *T. parva* Muguga sequence but could be assembled *de novo*.

### Southern blot analysis of recombinant MugugaUganda using telomeric probes

**Figure 2:**
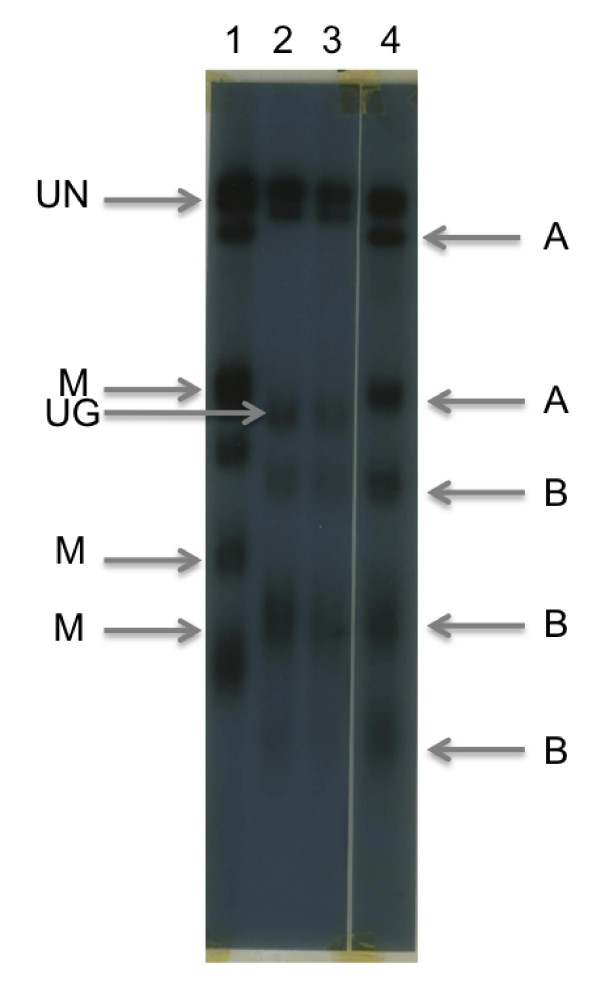
**Southern blot analysis of DNA from the MugugaUganda recombinant clone and parental Muguga and Uganda clones using a telomeric DNA probe.** A blot of *Eco*RI-digested *T. parva* DNA that was size fractionated through a 0.8% agarose gel and transferred onto a Hybond N Membrane is depicted. The blot was hybridised with a telomeric probe [48] and washed in 2X SSC at 60**°**C for 1 hour. The lanes are (1) *T. parva* Muguga cloned piroplasm DNA (1 ug), (2) *T. parva* Uganda schizont-infected lymphocyte DNA (20ug), (3) *T. parva* Uganda schizont-infected lymphocyte DNA (1ug) and (4) *T. parva* MugugaUganda recombinant clone piroplasm DNA (1ug). On the right hand side of the blot, the letter ‘A’ indicates two size polymorphic *Eco*RI restriction fragments of presumptive *T. parva* Muguga parental origin and the letter ‘B’ indicates three fragments of presumptive *T. parva* Uganda parental origin in the recombinant clone. Three telomeric fragments of *T. parva* Muguga parental origin that are absent from the recombinant parasite clone are indicated by an arrow on the left hand side of the blot, highlighted by the letter M. One telomeric fragment of *T. parva* Uganda origin is also inidicated by an arrow with the legend UG. Large *Eco*RI fragments that were not polymorphic using size fractionation through 0.8% agarose are indicated by an arrow and the letter UN on the left hand side of the blot. There were no detectable fragments that were clearly unique to the recombinant parasite.

The analysis of the MugugaUganda recombinant clone generated in this cattle co-infection and tick pick-up experiment revealed a mixed genotype by Southern blotting using a telomeric probe (see Methods). The presence of two fragments of Muguga origin (Figure 2, lane 4, highlighted by the letter A) and three smaller fragments of Uganda origin (Figure 2, lane 4, marked by the letter B; these smaller fragments are smeared presumably due to variation in the length of the terminal telomeric repeats) was detected. The parental origin of the other three fragments is ambiguous and we presume that they are in the large DNA fragments at the top of the gel that are not fully resolved (indicated by an arrow and the letters UN). There were three fragments of Muguga origin (indicated in Figure 2, lane 1, by an arrow and the letter M) and one of putative Uganda origin (indicated in Figure 2, lane 2, by an arrow and the letter UG) that were absent from the MugugaUganda recombinant (Figure 2, lane 4). There was no evidence found for novel fragments within the MugugaUganda recombinant and for major rearrangements adjacent to the telomeres, as has been demonstrated to occur in *P. falciparum*[22]. All five polymorphic telomeric *Eco*R1 fragments were identical in size to either the Muguga or Uganda parents, consistent with simple telomeric structure of *T. parva* that lacks major tracts of telomere-associated simple repeat sequences [1].

### Identification of SNP markers and parental alleles

SNPs in the two parental and two recombinant progeny clones were first identified by mapping the 454 reads to the Muguga reference genome. The *de novo* assemblies were also aligned with the Muguga genome with the aim of identifying SNPs in regions where the sequences were too divergent for mapping. Of regions unmapped in the Muguga genome, approximately 60% of the nucleotides remained unaligned (Table 3). Many of the genes that fall within these regions overlap with 27 genetic loci encoding *T. parva* antigens or multi-copy gene families that are likely to be under selection for diversification, such as the sub-telomeric *Sfi*I ST and SVSP families, the CD8+ target antigen Tp2, and the hyper-variable Tpr locus (Additional file 1: Figure S5). Others were either hypothetical proteins with unknown functions or contained low complexity repetitive sequences (Additional file 2: Table S3).

**Table 3:** **Unmapped and unaligned regions in *T. parva *Muguga genome by pair-wise comparisons to the four genomes sequenced in this study**

	**Marikebuni**	**Uganda**	**MugugaMarikebuni**	**MugugaUganda**
Unmapped bases ^1^	209716	943493	186820	165122
Unmapped and unaligned bases (% of unmapped bases)^2^	124013 (59%)	548856 (58%)	66465 (35%)	111753 (67%)
Number of genes with unmapped and unaligned bases	59	37	29	57

^1^ Unmapped by 454 reads using gsMapper.

^2^ Unmapped by 454 reads using gsMapper, unaligned by *de novo* assembled contigs using MUMmer.

**Figure 3:**
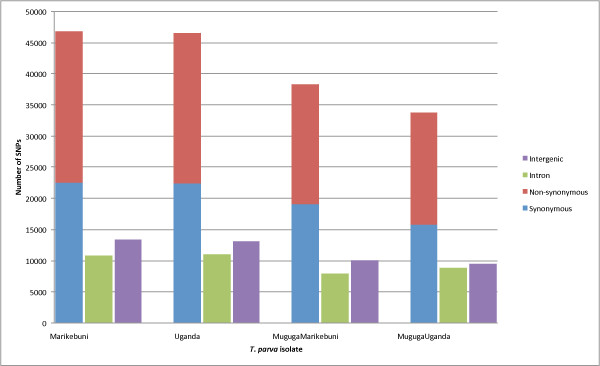
**Summary of single nucleotide polymorphisms in sequenced *T. parva *strains. ** Different coloured bars show the distribution of different SNP categories in each strain.

In total, 70977, 70608, 56378, and 52226 SNPs were identified in Marikebuni, Uganda, MugugaMarikebuni and MugugaUganda, respectively (Table 4). In all four isolates more than 65% of the SNPs were found in exons, between 14% and 17% of the SNPs were in introns while 18-19% of SNPs were in intergenic regions (IGR) (Figure 3). The proportions of SNPs observed in each category correspond to the proportions of these categories found in the genome [1,23]. Within exonic SNPs almost equal proportions of synonymous and non-synonymous SNPs were observed for all isolates.

**Table 4:** **Total number of SNPs in *T. parva *parent and progeny genomes**

**Strain**	**Total number of SNPs**	**Number of SNPs inferred from reference assemblies**	**Number of SNPs inferred from*de novo*assemblies**
Marikebuni	70977	68036	2991
Uganda	70608	67333	3275
MugugaMarikebuni	56378	53416	2962
MugugaUganda	52226	51701	525

**Figure 4:**
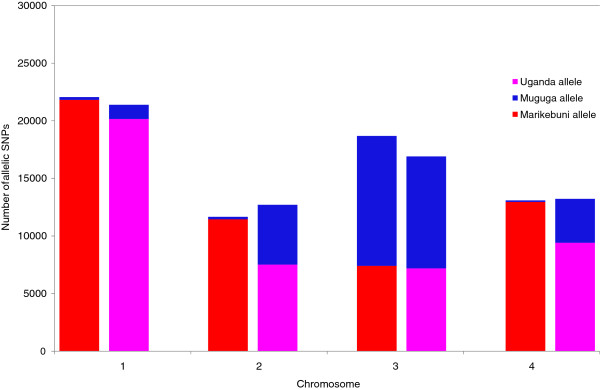
**Allelic SNP distribution in progeny strains MugugaMarikebuni and MugugaUganda.** In both progeny strains, all chromosomes except chromosome 3 had predominantly non-Muguga alleles. MugugaMarikebuni is depicted in the left and MugugaUganda in the right bars for each individual chromosome.

By combining the SNP markers of the parent and the corresponding progeny strain, we were able to genotype 65531 SNP markers in MugugaMarikebuni, and 64244 SNP markers in MugugaUganda (Additional file 3: Table S5 and Additional file 4: Table S6). 5446 Marikebuni and 6364 Uganda markers were not typed in the corresponding progeny strain due to lack of confident consensus calls in the progeny strain. High confidence non-parental SNPs were retrieved in the progenies – 734 (1.3%) and 589 (1.13%) in MugugaMarikebuni and MugugaUganda, respectively. A majority of these non-parental SNPs were observed as alleles of minor frequencies (allele frequency<50%) in its non-Muguga parental counterpart (data not shown) as would be expected from an infection of non-clonal parental strains. These SNPs were excluded from further analyses. The average distance between adjacent markers that could be genotyped in both progeny strains was 127 bp. This resolution is over 25 times higher than in the recently published *P. falciparum* recombinant map [20] and more than 800 times higher than in the current *T. parva* genetic map [10]. The level of sequence coverage here was similar to that in the *P. falciparum* study, suggesting that SNP numbers between the *T. parva* parents were much greater than in *P. falciparum*. In both progeny strains, the majority of the SNP alleles on 3 of the 4 chromosomes (numbers 1, 2 and 4) were inherited from the non-Muguga parent. For example, in the MugugaMarikebuni clone, only 1-2% of the SNP loci on chromosomes 1, 2 and 4 had Muguga alleles. In MugugaUganda, the bias towards Uganda alleles was less prominent - 5%, 41% and 29% loci had Muguga alleles in chromosomes 1, 2 and 4, respectively (Figure 4). Chromosome 3 had inherited more *T. parva* Muguga-derived alleles (~60%) than non-Muguga alleles (~40%) in both MugugaMarikebuni and MugugaUganda. Southern blot data supported this analysis by indicating that the tandemly arrayed Tpr locus located on Chromosome 3 was similar to that of Muguga (Bishop and Morzaria, data not shown).

### Detection of crossover events

**Figure 5:**
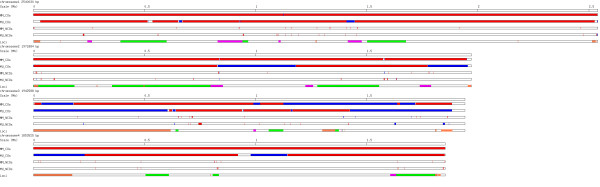
**Distribution of crossover (CO) and non-crossover (NCO) recombination events in progeny clones MugugaMarikebuni (MM) and MugugaUganda (MU).** Chromosomes 1 to 4 are lined up from top to bottom. The length of each chromosome was scaled to the real genomic size at the ratio of 1 pixel per 1000 nucleotides. In tracks of CO events (labelled as “MM_COs” and “MU_COs”), blue bars represented sequences originated from Muguga, red bars Marikebuni or Uganda. Each colour transition corresponds to a CO breakpoint. Empty regions represent unmapped regions, regions without SNP markers, or regions with such frequent allele changes that their origins could not be clearly defined (such as regions of complex gene conversions). The “NCOs” tracks (labelled as “MM_NCOs” and “MU_NCOs”) show positions of NCO events, with gene conversions to Muguga alleles in blue and to non-Muguga alleles in red. In the track labelled as “Loci”, the known recombination hot spots and cold spots [10] are highlighted as magenta bars and green bars, respectively. Positions of the 27 loci encoding products previously known as immune-relevant [10] are shown as orange bars.

**Figure 6:**
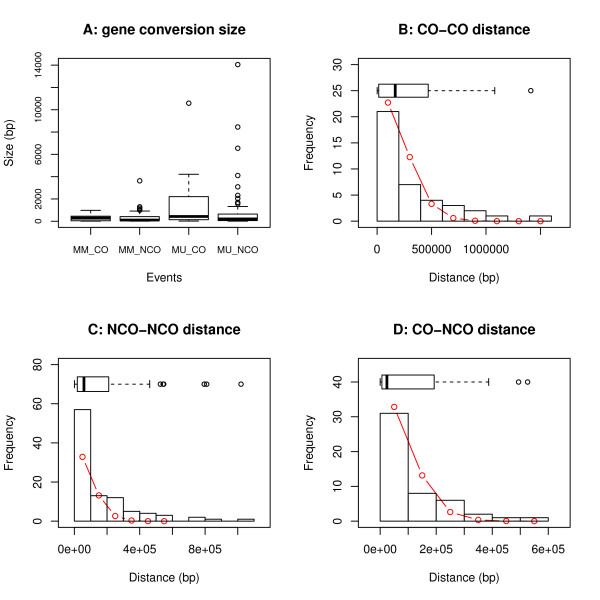
**Distributions of CO and NCO sizes (A), distance between COs (B), distance between NCOs (C), and distance between COs and NCOs (D).** The sizes of gene conversion tracks associated with COs and NCOs were not significantly different (**A**). Sizes were defined by the closest markers where the defined allele changes were observed (Additional file 14: Figure S7, materials and methods). The ends of the whiskers represent 1.5 interquartile range of the lower/upper quartile. The distributions of all three types of distance fit well with Poisson distributions (**B**, **C**, **D**). The red circles and lines represent expected Poisson distributions that fit to the datasets, as determined by the method of least squares. The R square values were 0.93295, 0.93026, and 0.95587, respectively. Distance values were calculated based on the start positions of consecutive breakpoints.

In the current study, we genotyped one MugugaMarikebuni and one MugugaUganda recombinant clone using more than 64 thousand markers. A sliding window of 15 contiguous SNP markers [16,20] was implemented for identification of COs. Fifteen and 24 CO breakpoints were identified in MugugaMarikebuni and MugugaUganda, respectively (Figure 5). The average distance between COs detected was 312 Kb, with a median distance of 156 Kb (Figure 6B). This can be compared to the previously determined *T. parva* linkage map, where the median number of COs per clone was 13 (ranging from 7 to 20, Additional file 5: Figure S1) and the average and median distance of COs were 463 Kb and 326 Kb, respectively. The much higher marker density achieved in our study allowed for the detection of more COs, especially COs separated by shorter distances (Figure 5). Two MugugaMarikebuni and four MugugaUganda CO breakpoints fall within previously defined high frequency recombination sites, whilst one MugugaMarikebuni and three MugugaUganda CO breakpoints overlapped with sites previously defined as non-recombinogenic [10]. Two MugugaMarikebuni and two MugugaUganda COs occurred within the sub-telomeric regions. These were not detectable in the earlier study due to the lack of VNTR marker coverage in those regions (Additional file 5: Figure S1).

The high marker density also allowed us to study CO associated gene conversions (Additional file 6: Table S1). In MugugaMarikebuni, eight (53%) of the CO breakpoints were simple breakpoints, where one parental allele changed into the other parental allele without any associated gene conversion. The other seven were complex breakpoints, associated with conversion tracts containing frequent allele changes. The length of the gene conversion tracks ranged from 7 bp to 1 Kb, with a median length of 277 bp (Figure 6A). In MugugaUganda, 11 (46%) were simple breakpoints and 13 (54%) were complex breakpoints. The median length of gene conversion tracks was 426 bp (with a size range from 17 bp to 11 Kb) (Figure 6A). In both progeny strains, the median length of the conversion tracks accompanying CO breakpoints was much shorter than the observed length of 2 Kb in yeast [17,24].

The percentage of complex breakpoints (about 50%) was higher to those reported for *P. falciparum* recombinants (33%, [20]) and yeast (11.5%, [17]). Such complex gene conversion tracks associated with COs have been predicted to result from the resolution of a double Holliday junction because of multiple distinct hetero-duplexes in a single CO event [25]. Mismatch repair alternating between conversion and restoration could also lead to formation of such complex gene conversion tracks [17]. This high fraction of COs accompanied by gene conversions could contribute to the diversity and evolutionary success of *T. parva*, by not only redistributing existing genetic variations, but also by changing allelic frequencies.

In MugugaUganda, each chromosome had at least one CO event, which is consistent with the expectation of one obligate CO per pair of homologous chromosome [26]. In MugugaMarikebuni, no CO events were detected in chromosome 4. Chromosomes without detectable COs were also observed amongst the 35 MugugaMarikebuni recombinant parasite clones typed with the VNTR markers (Additional file 5: Figure S1). This might be due to the fact that the obligate CO on chromosome 4 could have been formed on the sister chromatid not analyzed here.

### Effects of detection methods on crossover identification

To investigate the effect of the method employed for crossover identification, two sliding window methods to determine COs were implemented and applied for both progeny strains. One method varied the number of SNPs (Additional file 7: Figure S2 A) and the other method varied the physical distance (Additional file 7: Figure S2 B). The number of SNPs (7, 11, 15, 19, and 23) selected for each window size has been used in a similar study [16]. The physical distances (1 to 6 Kb) that were calculated based on the mean marker distance of 127 bp, roughly corresponded to the average distances between 7, 15, 23, 31, 39, and 47 SNPs, respectively.

In both methods, smaller window sizes identified more COs, and the extra COs tended to be smaller. For example, when the sliding window size was decreased to seven contiguous markers, one CO breakpoint of 1 Kb was detected on chromosome 4 at position 1.797 Mb in MugugaMarikebuni, which was not identified using larger window sizes. On chromosome 2 in MugugaMarikebuni, the 1 Kb window also detected two COs, which became undetectable when the window size increased. Counts and positions of major CO events remained the same for both methods, although the sensitivities of the two methods seemed to vary for small COs. For example, both the 15 SNPs and the 2 Kb distance windows scanned through the chromosomes with an average resolution of 2 Kb, the window of 2 Kb called smaller COs in both progeny strains on most chromosomes compared to the 15 SNP window. Also, the estimated sizes of the breakpoints obtained from these two methods were not always identical. For example, CO breakpoints on chromosome 1 at positions around 1.1 Mb in MugugaMarikebuni were detected by all window sizes. The breakpoint sizes calculated by the SNP method were too short to be visible on graphical plots, while those calculated by the method of physical distance were large enough to be visible in the plots of the same resolution (Additional file 7: Figure S2). On chromosome 2 in MugugaMarikebuni, the opposite was observed; here the breakpoints became visible with the SNP method but not with the physical distance scanning.

The differences between the two methods can largely be explained by the variation of SNP densities along the chromosomes. Windows of SNPs would scan through highly variable regions with shorter physical distance, while windows of physical distances would scan through such regions with higher number of markers. Consequently, in regions with higher marker density, the SNP method would detect smaller COs and in regions with lower marker density the distance method would annotate smaller COs. In our study, the distance method identified higher numbers of smaller COs than the SNP method, suggesting that regions of lower marker density were more abundant in our dataset than regions of higher marker density. We decided to use the SNP method using 15 markers for further analysis for the following practical reasons. First, the method has been applied in a similar study in *P. falciparum*[20] therefore allowing a direct comparison of the results between the two species. Second, we wanted to minimize detection of small COs as our study design did not make such calls with high confidence. Ideally, COs would be events where nucleotide sequences change from parent one (p1) to parent two (p2) in one meiotic product and from p2 to p1 in another at similar positions. An event where such a change in nucleotide sequence occurs only in one meiotic product is a non-crossover (NCO) [17,18]. However, due to the technical challenges involved in generating recombinant clones, in the current study only one out of four meiotic products was sequenced for each pair of parental clones. Consequently, we cannot exclude the possibility that some of the COs could be tracts of NCOs. For example, small COs found on chromosome 1 and chromosome 2 in MugugaMarikebuni (Figure 5, Additional file 6: Table S1) might actually be NCOs, considering the very low number of Muguga alleles observed on these two chromosomes (Figure 4).

### Detection of non-crossover events

The high density of markers also allowed us to characterize non-crossover (NCO) gene conversions. Fifty-two and 56 NCO events were identified in MugugaMarikebuni and MugugaUganda, respectively (Figure 5). In both progeny clones, all chromosomes carried one or more NCOs. In total, we identified 15 COs and 52 NCOs for MugugaMarikebuni and 24 COs and 56 NCOs for MugugaUganda. This sum of COs and NCOs (67 and 90) was lower than the estimate of 140–170 double-strand breaks (DSBs) per meiosis in yeast, but higher than the 49 described in *P. falciparum*[20]. Yeast and *P. falciparum* are not suitable comparators for *T. parva* as although the genome sizes of yeast and *T. parva* are similar (12 Mb vs. 8.3 Mb) yeast has more chromosomes (16 vs. 4). Additionally *P. falciparum*, which is pylogenetically closer to *T. parva*, has a larger genome (23.3 Mb vs. 8.3 Mb). The total recombination events we observe is likely to represent a minimal estimate of the true total. The stringent definition of NCO applied here may have missed conversion events involving less than three SNP markers, or those that may have resulted in restoration of the original alleles. NCO signatures containing a complex pattern of genotype changes were also not included in the totals. This includes several long and complex gene conversion signatures observed in sub-telomeric regions in both progeny clones (Figure 5). By analyzing only one meiotic product out of the four, theoretically half of the COs and two thirds of the NCOs would have been missed. We were not able to differentiate long NCOs (i.e. NCOs involving 15 markers or more) from COs either, although this would not affect the total. As discussed earlier, genotyping of all four meiotic products will be needed to address these issues [18].

Compared to the length of CO-associated gene conversion signatures, the length of NCO conversion tracks was not significantly different (Mann–Whitney Wilcoxon test, P-value = 0.3297, Figure 6A), which deviates from previous observations in yeast and humans [27,28]. The median length of the NCO gene conversion track was 108 bp and 207 bp in MugugaMarikebuni and MugugaUganda, respectively. This is considerably shorter than estimates from *Saccharomyces cerevisiae*[17,29] but similar to those in *Homo sapiens*[27]. In MugugaMarikebuni, seven NCOs overlapped with the previously defined CO hot spots and eight with putative non-recombinogenic regions. In the MugugaUganda progeny clone considerably more NCOs overlapped with the known CO cold spots compared to hot spots (12 vs. 2). In both strains, NCOs were distributed rather uniformly across each chromosome, including the sub-telomeric regions. However, clusters of NCOs were also observed such as in the sub-telomeric region of chromosome 2.

### Crossover and non-crossover interference

The distance between recombination events could reflect interference. In yeast CO-CO and CO-NCO interference has been reported [17,18]. We analyzed the distributions of distances between CO-CO (Figure 6B), NCO-NCO (Figure 6C), and CO-NCO (Figure 6D) events. All three distributions were consistent with the Poisson distribution (Figure 6B, C and D), suggesting there was no measurable interference among the recombination events. However, more measurements would be required for a thorough study of interference among COs and NCOs.

### Effects of marker density on detections of crossover and non-crossover

**Figure 7:**
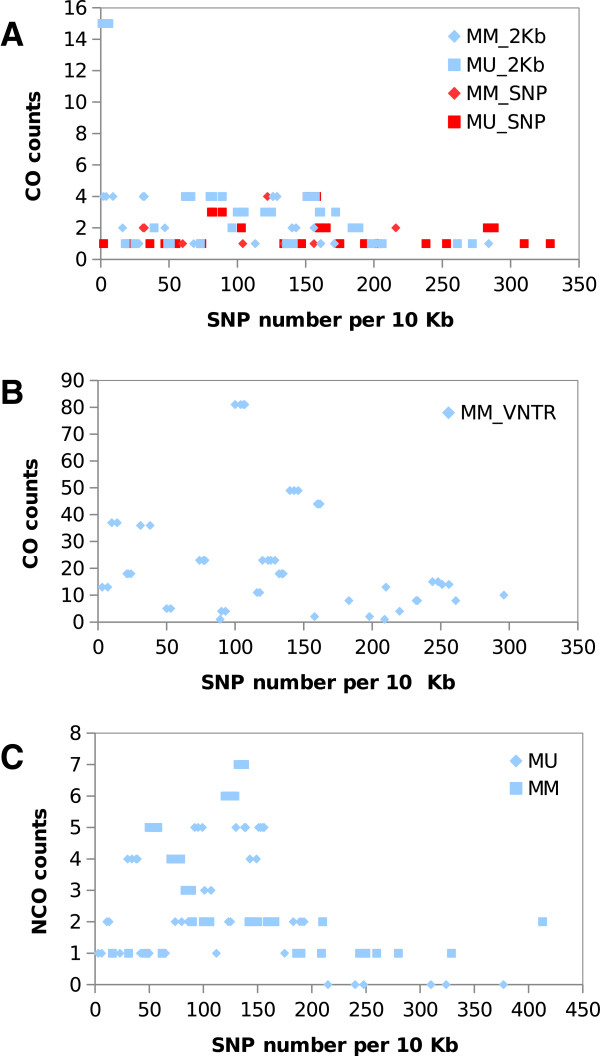
**Distribution of recombination events among regions of different SNP densities.** Recombination events in the recombinant clones show a negative correlation with SNP densities. (**A**) COs found in our study. (**B**) COs identified by VNTR typing and reported by Katzer F. et *al*. [10]. (**C**) NCOs found in our study. MM_SNP: SNP typing of MugugaMarikebuni. MU_SNP: SNP typing of MugugaUganda. MM_VNTR: VNTR typing results of MugugaMarikebuni.

To test if SNP marker density had an effect on recombination frequency, we calculated marker densities along each of the chromosomes using a 10 Kb window, and graphically plotted the total number of COs (Figure 7A) against their corresponding marker density values. Similar plots were generated for COs identified using VNTR by Katzer F. et al. [10] (Figure 7B) and NCOs found in this study (Figure 7C). We found a reduced frequency of COs in regions of high marker densities and the methods used to identify COs had little effect on this correlation. Similar observations that sequence divergence between parental strains adversely affects CO frequencies have been reported in yeast [30].

### Genomic locations of recombination breakpoints

The location of breakpoints differed between COs and NCOs. Most of the NCO breakpoints (~65%) occurred within genes, while ~15% were either located in IGR or overlapped with the gene-IGR boundary (Additional file 8: Table S2). In contrast, a majority (70-80%) of the CO breakpoints overlapped with the gene-IGR boundaries, only five (33%) MugugaMarikebuni and three (13%) MugugaUganda CO breakpoints fell within genes. Most DSBs are known to occur in the promoter regions [31,32]. Classical eukaryotic promoter elements are thought to be absent in apicomplexan species, and it appears that regulatory motifs located in the IGR are responsible for transcription regulation [33]. Studies in yeast showed that > 84% of DSBs were located in promoter regions [17,31,32]. In comparison, a significantly smaller proportion (P-value = 0.0001, Fischer’s exact test) of CO and NCO breakpoints in MugugaMarikebuni - 47% (7) of COs, 3% (5) of NCOs - and MugugaUganda - 42% (10) of COs, 15% (8) of NCOs - overlapped with the putative promoter regions. Interestingly, of the breakpoints that overlapped with promoter regions, 25% (3) and 28% (5) in MugugaMarikebuni and MugugaUganda, respectively, contained four of the five most over-represented regulatory motifs in *T. parva* identified by Guo and Silva [33].

### Correlation of recombination events with chromosome size

In the two recombinant clones analyzed, the number of COs and NCOs did not correlate with chromosome sizes (Additional file 9: Figure S6). There were differences in the distribution of COs among the four chromosomes in the two recombinant clones. For example, on chromosome 1 of MugugaMarikebuni, there was one CO per 1.3 Mb, while it was one per 0.3 Mb in MugugaUganda. Distributions on chromosomes 2 and 3 were similar in both progeny strains, with one CO per 0.5 Mb on chromosome 2 and 0.3 Mb on chromosome 3, respectively. Chromosome 4 showed the lowest number of COs in both clones. Analysis of more recombinants would be essential to substantiate these results and to identify any correlations, if they exist.

### Intra-species polymorphism analysis

Although *T. parva* genes potentially under positive selection have been previously identified by inter-species polymorphism analysis [34], genome wide intra-species polymorphism analysis has not been performed systematically. We aligned 4076 *T. parva* Muguga mRNA sequences to genome drafts of Marikebuni, MugugaMarikebuni, MugugaUganda and Uganda. About 3600 mRNA sequences could be fully aligned and were subjected to Ka/Ks analysis (Additional file 10: Figure S3). The mean value of Ka/Ks ratio was about 0.7 (Additional file 11: Figure S4). In total, we identified 81 genes with Ka/Ks ratio above 1 among the four strains (Additional file 12: Table S4). Comparison of these genes to the eukaryotic orthologous groups (KOGs) did not find any functional category that was enriched within these genes potentially under positive selection. The BLAT alignment method of identifying gene homologues in the four strains limits our analysis to less diverged genes. Genes with highly divergent sequences were not subjected to Ka/Ks calculation as a full-length sequence alignment with their respective Muguga counterpart was not obtained. Amongst these diverse genes is the CTL antigen, Tp2 [35] that was found to be under positive selection in cattle and buffalo isolates [12]. Pain et *al*. [23] predicted a total of 3265 orthologous genes between *T. parva* and *T. annulata* genomes. Inter-species Ka/Ks analyses of these orthologous genes produced highest Ka/Ks values for predicted merozoite surface proteins and macroschizont proteins without predicted membrane retention motifs potentially secreted into the leukocyte cytosol. These proteins could be involved in immunological interplay between host and parasite. Amongst genes without KOG matches but with Ka/Ks ratio above 1 was TP02_0174, which has been annotated as a putative sporozoite surface protein. Its orthologue in *T. annulata* has been characterized as sporozoite and macro-schizont gene 2 (Spm2) expressed in sporozoites, macro-schizont-infected leucocyte and piroplasm stages. A monoclonal antibody specific for Spm2 blocked partial invasion of sporozoites to host cells indicating that this protein could play a role in protective immune responses [36].

## Conclusions

Roche 454 GS FLX Titanium chemistry was used to sequence the 8.3 Mbp genomes of two haploid progeny clones from an experimental *T. parva* co-infection in cattle and two of the parent isolates. All four strains were genome typed by comparing the 454 reads with the *T. parva* Muguga reference genome, which represented the other parent of both recombinant clones. Our data demonstrate that the A-T rich genome of *T. parva* can be reliably sequenced and assembled with 454 technology. The panel of genome-wide SNPs established can be used for molecular epidemiology studies in the future, particularly to trace potential new alleles introduced in *T. parva* endemic regions by applying the live vaccination method of “infection and treatment” [37]. Additionally, the list of genes with high Ka/Ks values by intra-species comparisons can be further explored for potential functional implications. The high resolution mapping of COs and NCOs on all four chromosomes showed patterns distinct from *P. falciparum*, yeast and humans. The high number of gene conversion events found to be associated with recombination between different *T. parva* genotypes was not previously known. This observation, together with the relatively high crossover frequency reported previously[10], indicates that meiotic recombination plays a very important role in generation and distribution of genetic diversity in *T. parva* population, which is essential for the evolutionary success of the pathogen. The high multiplicity of infection in both cattle and the ancestral wildlife host, cape buffalo, suggests that recombination processes may occur frequently under natural conditions, thus contribute to the high level of polymorphism observed in *T. parva* in the field.

## Methods

### Parasite parental and recombinant clones

This study describes the whole genome sequencing of two cloned *T. parva* genotypes that were originally described by Morzaria et *al*., [38], *T. parva* Marikebuni (clone stabilate 3292) and *T. parva* Uganda (clone stabilate 3645) and two recombinant parasite clones derived thereof [39]. Additional details regarding the generation of the clonal recombinant parasites are provided herein. All protocols and experimental procedures relating to *in vivo* infections used to generate recombinant parasite clones and piroplasms for extraction of DNA for genome sequencing were first evaluated by the ILRI Animal Care and Use Committee (IACUC), which ensures that all animal experiments adhere to international standards.

### Animal infection and recovery of recombinant parasite clones

Cattle were inoculated with two different *T. parva* stocks, either Muguga and Marikebuni or Muguga and Uganda. From these co-infected animals, tick pick up experiments were performed and sporozoites were isolated from these tick salivary glands. Acini containing putatively recombinant parasites were identified using PCR amplification with conserved Tpr locus primers, followed by hybridization with stock-specific oligonucleotide probes designed from the Tpr locus [40,41]. The hybridization analysis indicated that 38% of these acini represented mixtures containing genetic material from both parents [5,39]. Isolated sporozoites from single infected acini with mixed infections were then used to infect peripheral blood mononuclear cells *in vitro* at very high dilutions to maximise probability that the cells were infected with a single sporozoite. The resulting schizont transformed cell lines were then twice cloned by limiting dilution to arrive at a clonal progeny [38]. The schizont-infected cell lines containing the putative recombinants were used to infect the autologous animals from which the peripheral blood mononuclear cells were obtained originally and the piroplasms were isolated. A detailed protocol describing the complete procedure for isolation of clonal recombinant parasites is provided in the Additional file 13: Supplementary material and methods.

### Piroplasm purification, DNA preparation and southern blotting

Piroplasm in suspension was lyzed by mixing gently in the presence of 10mM Tris-Cl, pH7.4, 100mM EDTA, 50ug/ulRNase A and 0.5% SDS and incubated at room temperature (23°C) for 10 min. Then Proteinase K is added to the mixture at 100ug/ml, mixed and incubated at 56°C for 4 hours. NaCl is added to 100mM and the mixture is extracted with one volume water-saturated phenol-chloroform-isoamyl alcohol (25/24/1) by shaking gently overnight at 4°C. After centrifugation at 5,000 rpm at 4°C for 20 min, the upper aqueous phase containing the DNA is transferred in a fresh tube and 2 volume of absolute ethanol chilled at −20°C is gently layered on top of the aqueous phase and the DNA is precipitated by returning the tube gently to mix. The supernatant is decanted and the DNA pellet is washed trice with large amount of 70% ethanol, air-dried then re-dissolved in 10mM Tris-Cl, pH 7.4, 1mM EDTA (TE) at 4°C overnight. Southern blotting onto Hybond N filters, probe labeling and hybridization was performed exactly as described previously [21].

### Genotyping by whole genome sequencing

Genomic DAN samples were fragmented and sequenced with Roche 454 Titanium chemistry according to manufacturer’s protocols. All 454 read files have been submitted to NCBI Sequence Read Archive (SRA055319). Single nucleotide polymorphisms (SNPs) in the two parent and two recombinant progeny isolates were first called by mapping 454 reads to the Muguga genome using the Roche 454 Software GS Reference Mapper (Version 2.5.3). The high confidence SNP calls made by the software were further filtered to include only SNPs with depth of more than six non-duplicate reads and allele frequency over 50%. SNPs were also called independently by aligning the *de novo* assembly of each strain with the Muguga genome using MUMmer, which were filtered using the following rules: 1) supported by at least six non-duplicate reads; 2) had consensus quality score of 30 or above; 3) flanking regions (15 bp up and downstream) had less than 20% of bases whose consensus quality scores were less than 30. Insertions and deletions were not considered. One of the shortcomings of the 454 technology is the high error rate in calling homopolymers longer than 8 bp, which could reflect as erroneous non-synonymous mutations arising from frameshifts. SNPs within homopolymers were filtered out. Finally, the SNP sets were combined and those that fell into repetitive regions of Muguga genome were excluded as the final filtering step.

To determine if a SNP was inherited from Marikebuni/Uganda, MugugaMarikebuni and MugugaUganda progeny SNPs were compared with those of Marikebuni and Uganda parents, respectively. To identify alleles inherited from Muguga in the two progeny strains, for each SNP locus in Marikebuni/Uganda, the consensus sequence of the corresponding progeny strain at the same location was checked. If the consensus sequence was the same as in Muguga genome, and was supported by at least three non-duplicate reads, a Muguga allele was called in the progeny strain at the given location.

### Detection of recombination events

For identification of COs, the sliding window method of 7 to 23 contiguous SNP markers [16,20] and the sliding window method of 1 to 6 Kb were implemented and compared. The sliding window of 15 contiguous SNP markers was selected for further analysis for the reasons explained in the Results section. For every marker locus, allele frequency was calculated using the sliding window. A CO breakpoint was defined as the region where the allele frequency transitioned from 100% of one parental allele type to 100% of the other parental allele type. NCO breakpoints were strictly defined as a region consisting of 3 to 14 contiguous markers with one parental allele type, flanked by longer stretches of markers with the other parental allele type. The distance between events was calculated as the distance between the start sites of two consecutive breakpoints. The sizes of breakpoints were maximal distances defined by the closest markers with defined allele frequency changes (Additional file 14: Figure S7, materials and methods), which can be over-estimations due to the limited SNP information [18].

### Identification of promoter regions

Promoter region boundaries were determined based on a previous study that identified regulatory motifs in *T. parva*[33]. They were positioned 300 bp upstream of the start position of genes in the reference Muguga strain [1]. For intergenic regions (IGR) shorter than 300 bp the entire IGR was considered as a promoter region. Regions upstream of genes that overlapped with another gene were ignored. Genes on each strand were treated separately. CO and NCO breakpoints that overlapped with the putative promoter regions were identified using the fjoin program [42].

### Intra-species polymorphism analysis

Full-length mRNA sequences from *T. parva* Muguga genome were aligned to both *de novo* and mapped assemblies using BLAT [43]. For every pair of fully aligned mRNA sequences, the Ka/Ks ratio, i.e. the ratio of the number of non-synonymous substitutions per non-synonymous site (Ka) to the number of synonymous substitutions per synonymous site (Ks), was calculated using Nei and Gojobori approximate method [44] implemented in the Ka/Ks Calculator [45]. Genes with Ka/Ks ratios above average and the corresponding Fisher exact test P-value below 0.05 were selected as genes potentially under positive selection. Gene sequences were compared to the eukaryotic orthologous groups (KOGs) [46] using Reversed Position Specific BLAST (RPS Blast) [47] for functional group classifications.

## Abbreviations

CO: Crossover; DSB: Double strand break; ECF: East coast fever; GS FLX: Genome sequencer FLX; IGR: Intergenic region; Kb: Kilo base pairs; Mbp: Mega base pairs; NCO: Non-crossover; SNP: Single nucleotide polymorphism; VNTR: Variable number tandem repeat.

## Competing interests

The authors declare that they have no competing interests.

## Authors' contributions

CD, RB, SM and WQ conceived the study, participated in its design and coordination and wrote the manuscript. SM and PS established the recombinant parasite clones. SM and RP supplied the genomic DNA for sequencing. LP sequenced all four strains with mate paired libraries. WQ designed the sequencing strategy, supervised SH, performed data analysis with SH, and drafted the manuscript. UC and EK sequenced Marikebuni with fragment reads, which were analyzed by UC, EK and ME in an earlier, separated report. All authors read and approved the final manuscript.

## Supplementary Material

Additional file 1: Figure S5 Unmapped and unaligned regions in the sequenced genomes. Chromosomes 1 to 4 are arranged from left to right. Strains are lined up from top to bottom, where the strain name is indicated at the beginning of each track. Positions of the 27 loci encoding products previously known as immune-relevant [10] are shown in orange using the “Loci” track. M: Marikebuni. MM: MugugaMarikebuni; MU: MugugaUganda. U: Uganda. Click here for file

Additional file 2: Table S3 Genes unmapped to *T. parva* Muguga reference genome in each strain. ‘M’ indicates mapped and ‘UM’ indicates unmapped in the strain. Click here for file

Additional file 3: Table S5 Genotyping results of MugugaMarikebuni. Click here for file

Additional file 4: Table S6 Genotyping results of MugugaUganda. Click here for file

Additional file 5: Figure S1 Comparison of crossover events detected using SNP markers with those identified using VNTR markers [10]. Chromosomes 1 to 4 are arranged from left to right. Identified CO events in each progeny strain are lined up from top to bottom, where the strain name is indicated at the beginning of each track. Colour transitions between blue and red indicate CO events. MugugaMarikebuni (MM) and MugugaUganda (MU) were the two recombinant clones analyzed in our study. All other clones were crosses of Muguga and Marikebuni, which were analyzed with VNTR markers as described in [10]. Click here for file

Additional file 6: Table S1 Crossover (CO) breakpoints and sizes of associated gene conversions (GC) in the progeny strains. Click here for file

Additional file 7: Figure S2 Detection of crossover events when varying the sliding window size. (A) Number of SNPs as the sliding window size. Tested sizes were 7, 11, 15, 19 and 23 SNPs, respectively. (B) Absolute physical distance as the sliding window size. Tested sizes were 1, 2, 3, 4, 5, 6 Kb, respectively. In both figures, chromosomes 1 to 4 are arranged from left to right. Identified CO events in each progeny strain using different window sizes are lined up from top to bottom, where the strain name and the window size are indicated at the beginning of each track. Color transitions between blue and red indicate CO events. Due to limited resolution, a few CO events of very short sizes are not visible, including the CO breakpoint of 1 Kb on chromosome 4 at the position of 1.797 Mb in MugugaMarikebuni, which was detected by the window size of 7 SNPs, as well as the CO breakpoints on chromosome 1 at positions around 1.1 Mb in MugugaMarikebuni, which were detected by all window sizes but only visible for windows of physical distances. MM: MugugaMarikebuni; MU: MugugaUganda. Click here for file

Additional file 8: Table S2 Non-crossover breakpoints in the progeny strains. Click here for file

Additional file 9: Figure S6 Number of recombination events by chromosome sizes. MM: MugugaMarikebuni. MU: MugugaUganda. CO: cross over event. NCO: non-cross over event. TOT: all recombination events (COs plus NCOs). Click here for file

Additional file 10: Figure S3 Distribution of Ka/Ks ratios by intra-species polymorphism analysis. Red bars represent histograms of Ka/Ks ratios. Blue curves are normal distribution curves fit to the corresponding histograms. The top four graphs were based on BLAT alignment of Muguga mRNAs to *de novo* assemblies, bottom four were to mapped assemblies. Click here for file

Additional file 11: Figure S4 Ka/Ks ratios by Fisher exact test P-values. Vertical lines represent the means of Ka/Ks ratios. Horizontal lines represent the cut-off P-value (0.05). The top four graphs were based on BLAT alignment of Muguga mRNAs to *de novo* assemblies, bottom four were to mapped assemblies. Click here for file

Additional file 12: Table S4 Genes potentially under positive selection by intra-species polymorphism analysis. Click here for file

Additional file 13 Supplementary information on Material and Methods. Click here for file

Additional file 1: Figure S7 Alleles around recombination breakpoints and definitions of breakpoint boundaries. One example for each breakpoint type was shown: (A) Simple CO breakpoints without associated gene conversions. Around these breakpoints, markers had the following characteristics: start with at least 15 continuous alleles of p1, followed by at least 15 continuous alleles of p2 (or 15 p2 alleles followed by 15 p1 alleles, for simplicity we use the p1 to p2 change as an example). The p1 allele frequencies would be 15/15, 14/15, 13/15, . . ., 1/ 15, 0/15 (which corresponded to a 15/15 p2 allele frequency). Thus the size of the breakpoint would be the length of the 15 p1 alleles plus the distance to the first p2 allele, as indicated by the yellow bars. (B) Complex breakpoints with gene conversions. Markers around these break points start with 15 continuous p1 alleles (p1 allele frequency of 15/15, followed by 1 to 14 p2 alleles, then 1to 14 p1 alleles, . . ., until it reaches a track of at lease 15 continuous p2 alleles so the p1 allele frequency reaches 15/15). (C) NCO breakpoints. NCO breakpoints were strictly defined as a region consisting of 3 to 14 contiguous markers with one parental allele type, flanked by longer stretches of markers with the other parental allele type. Muguga alleles are shown in red, while Uganda alleles in blue. Yellow bars indicate the boundaries of breakpoints, where the allele frequency changes from 100% of one parent to 100% of the other parent. For each marker, such allele frequency was calculated using the sliding window method described in materials and methods. Click here for file
