# Supplementary figures and images for: High-resolution genotyping and mapping of recombination and gene conversion in the protozoan Theileria parva using whole genome sequencing
Source: BMC Genomics. 2012 Sep 23;13:503. doi: 10.1186/1471-2164-13-503 (PMC3575351; doi:10.1186/1471-2164-13-503)

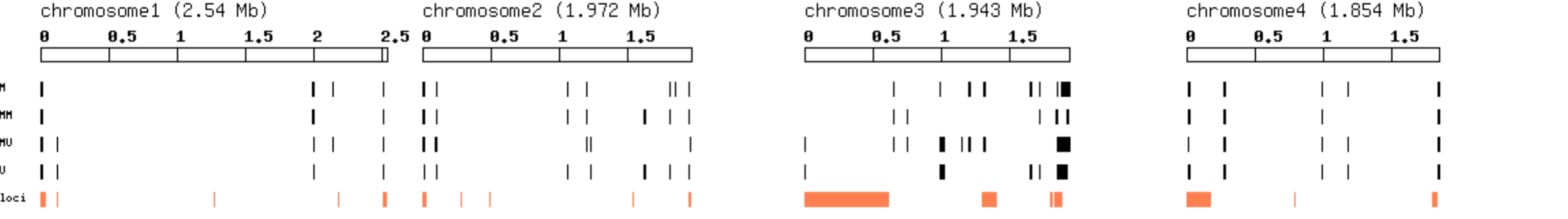

Supplement: Additional file 1: Figure S5 — Unmapped and unaligned regions in the sequenced genomes. Chromosomes 1 to 4 are arranged from left to right. Strains are lined up from top to bottom, where the strain name is indicated at the beginning of each track. Positions of the 27 loci encoding products previously known as immune-relevant [10] are shown in orange using the “Loci” track. M: Marikebuni. MM: MugugaMarikebuni; MU: MugugaUganda. U: Uganda. [file 1471-2164-13-503-S1.pdf]

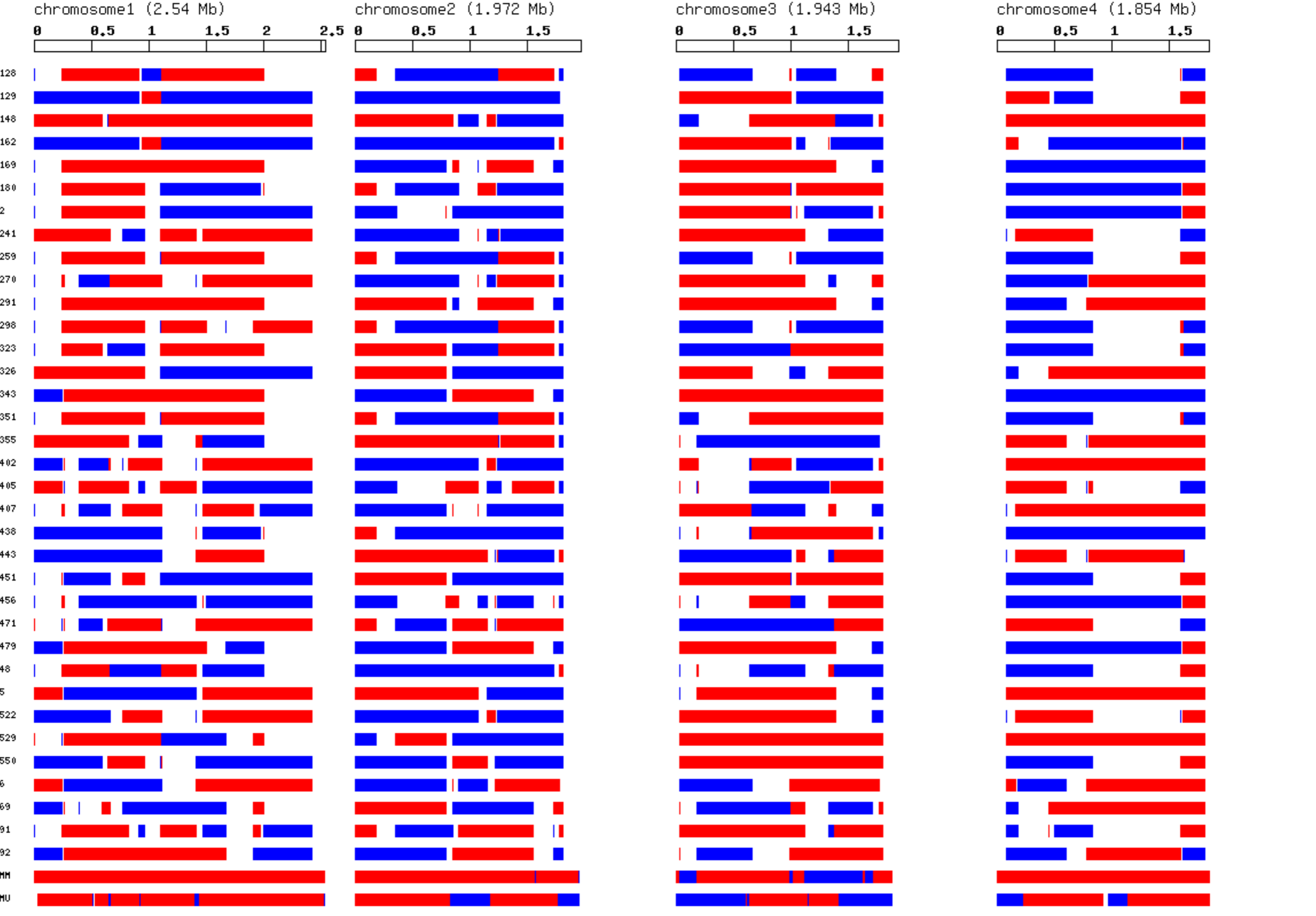

Supplement: Additional file 5: Figure S1 — Comparison of crossover events detected using SNP markers with those identified using VNTR markers [10]. Chromosomes 1 to 4 are arranged from left to right. Identified CO events in each progeny strain are lined up from top to bottom, where the strain name is indicated at the beginning of each track. Colour transitions between blue and red indicate CO events. MugugaMarikebuni (MM) and MugugaUganda (MU) were the two recombinant clones analyzed in our study. All other clones were crosses of Muguga and Marikebuni, which were analyzed with VNTR markers as described in [10]. [file 1471-2164-13-503-S5.pdf]

A

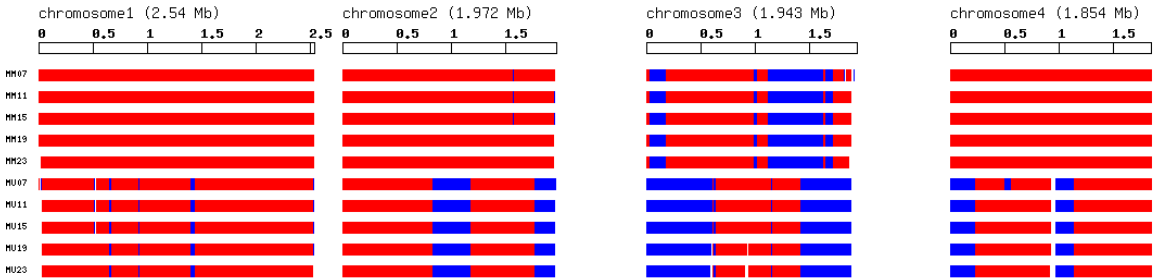

B

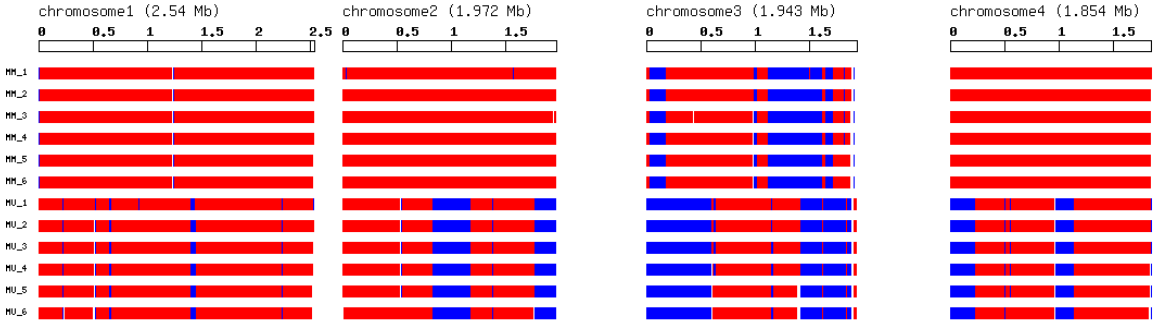

Supplement: Additional file 7: Figure S2 — Detection of crossover events when varying the sliding window size. (A) Number of SNPs as the sliding window size. Tested sizes were 7, 11, 15, 19 and 23 SNPs, respectively. (B) Absolute physical distance as the sliding window size. Tested sizes were 1, 2, 3, 4, 5, 6 Kb, respectively. In both figures, chromosomes 1 to 4 are arranged from left to right. Identified CO events in each progeny strain using different window sizes are lined up from top to bottom, where the strain name and the window size are indicated at the beginning of each track. Color transitions between blue and red indicate CO events. Due to limited resolution, a few CO events of very short sizes are not visible, including the CO breakpoint of 1 Kb on chromosome 4 at the position of 1.797 Mb in MugugaMarikebuni, which was detected by the window size of 7 SNPs, as well as the CO breakpoints on chromosome 1 at positions around 1.1 Mb in MugugaMarikebuni, which were detected by all window sizes but only visible for windows of physical distances. MM: MugugaMarikebuni; MU: MugugaUganda. [file 1471-2164-13-503-S7.pdf]

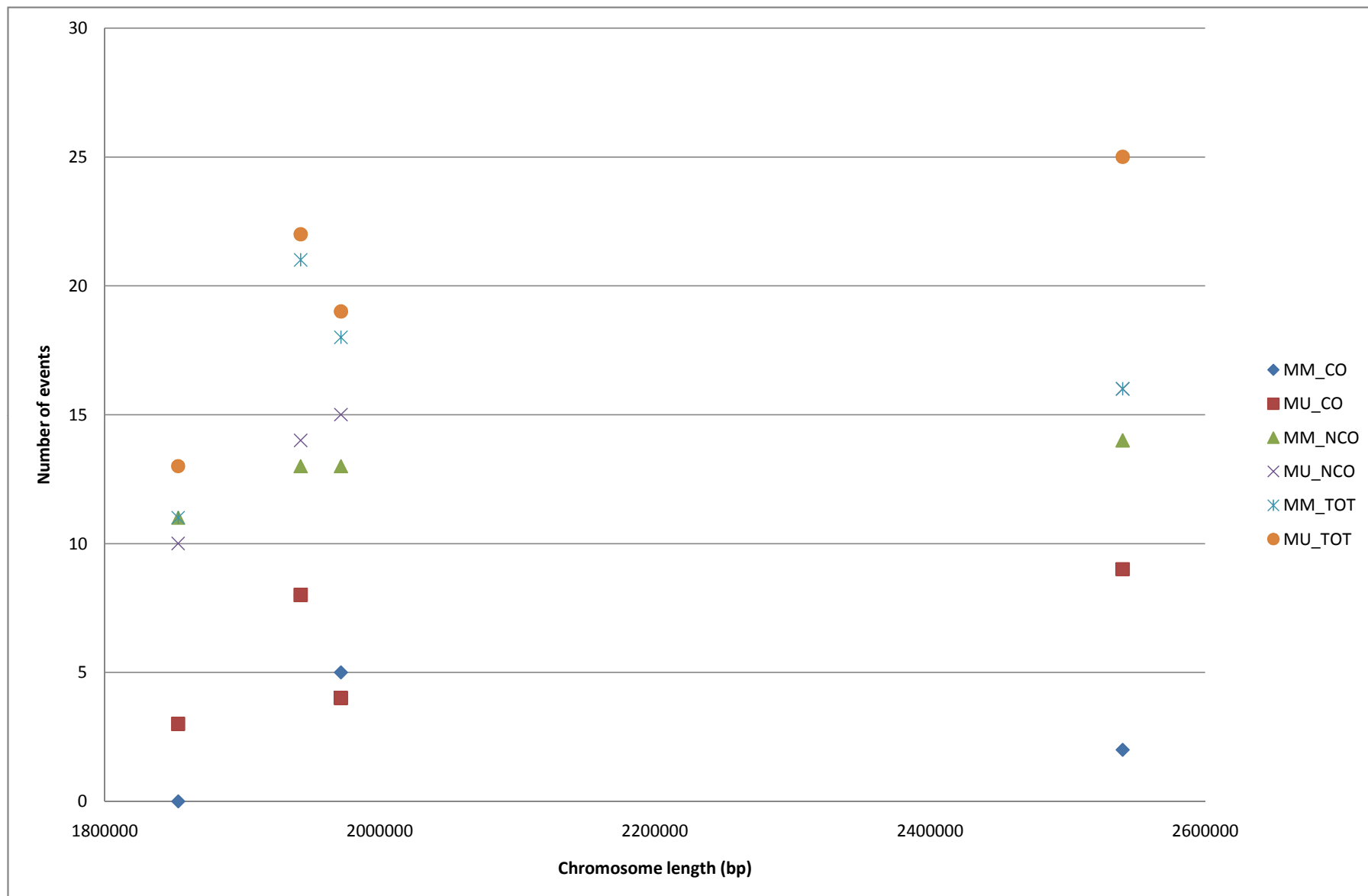

Supplement: Additional file 9: Figure S6 — Number of recombination events by chromosome sizes. MM: MugugaMarikebuni. MU: MugugaUganda. CO: cross over event. NCO: non-cross over event. TOT: all recombination events (COs plus NCOs). [file 1471-2164-13-503-S9.pdf]

(A) Simple CO breakpoint

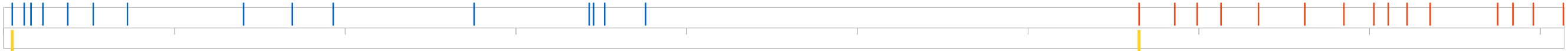

(B) Complex CO breakpoints

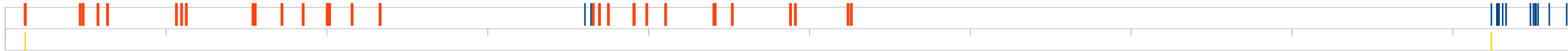

(C) NCO breakpoints

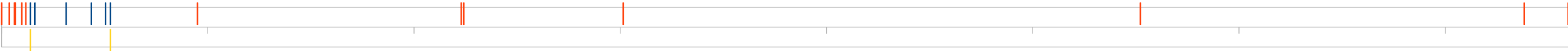

Supplement: Additional file 1: Figure S7 — Alleles around recombination breakpoints and definitions of breakpoint boundaries. One example for each breakpoint type was shown: (A) Simple CO breakpoints without associated gene conversions. Around these breakpoints, markers had the following characteristics: start with at least 15 continuous alleles of p1, followed by at least 15 continuous alleles of p2 (or 15 p2 alleles followed by 15 p1 alleles, for simplicity we use the p1 to p2 change as an example). The p1 allele frequencies would be 15/15, 14/15, 13/15, . . ., 1/ 15, 0/15 (which corresponded to a 15/15 p2 allele frequency). Thus the size of the breakpoint would be the length of the 15 p1 alleles plus the distance to the first p2 allele, as indicated by the yellow bars. (B) Complex breakpoints with gene conversions. Markers around these break points start with 15 continuous p1 alleles (p1 allele frequency of 15/15, followed by 1 to 14 p2 alleles, then 1to 14 p1 alleles, . . ., until it reaches a track of at lease 15 continuous p2 alleles so the p1 allele frequency reaches 15/15). (C) NCO breakpoints. NCO breakpoints were strictly defined as a region consisting of 3 to 14 contiguous markers with one parental allele type, flanked by longer stretches of markers with the other parental allele type. Muguga alleles are shown in red, while Uganda alleles in blue. Yellow bars indicate the boundaries of breakpoints, where the allele frequency changes from 100% of one parent to 100% of the other parent. For each marker, such allele frequency was calculated using the sliding window method described in materials and methods. [file 1471-2164-13-503-S14.pdf]
