# Supplementary material for: High-resolution genotyping and mapping of recombination and gene conversion in the protozoan Theileria parva using whole genome sequencing
Source: BMC Genomics. 2012 Sep 23;13:503. doi: 10.1186/1471-2164-13-503 (PMC3575351; doi:10.1186/1471-2164-13-503)
Supplement: Additional file 2: Table S3 — Genes unmapped to T. parva Muguga reference genome in each strain. ‘M’ indicates mapped and ‘UM’ indicates unmapped in the strain. [file 1471-2164-13-503-S2.doc]

**Supplementary Table 3. Genes unmapped to *T. parva*** Muguga reference genome in each strain. ‘M’ indicates mapped and ‘UM’ indicates unmapped in the strain.

| Gene | Marikebuni | Uganda | MugugaMarikebuni | MugugaUganda | Annotation |
| --- | --- | --- | --- | --- | --- |
| TP01_0001 | UM | UM | UM | M | hypothetical telomeric *Sfi*I fragment 20 protein 1 |
| TP01_0002 | UM | M | UM | M | hypothetical telomeric *Sfi*I fragment 20 protein 2 |
| TP01_0003 | UM | UM | UM | UM | hypothetical telomeric *Sfi*I fragment 20 protein 3 |
| TP01_0056 | M | UM | M | UM | Hypothetical protein, Tp2 |
| TP01_0966 | UM | M | UM | M | Hypothetical protein |
| TP01_0967 | M | UM | M | UM | Hypothetical protein |
| TP01_1042 | UM | M | M | UM | Hypothetical protein |
| TP01_1221 | UM | UM | UM | UM | Hypothetical protein |
| TP02_0002 | UM | M | UM | UM | Hypothetical protein |
| TP02_0006 | UM | UM | UM | UM | Hypothetical protein |
| TP02_0037 | M | M | M | UM | Hypothetical protein |
| TP02_0043 | UM | UM | UM | UM | Hypothetical protein |
| TP02_0527 | UM | UM | UM | M | Hypothetical protein |
| TP02_0592 | UM | M | UM | UM | Hypothetical protein |
| TP02_0608 | M | UM | M | UM | Hypothetical protein |
| TP02_0802 | M | UM | UM | M | Hypothetical protein |
| TP02_0896 | UM | UM | UM | M | Hypothetical protein |
| TP02_0911 | UM | M | M | M | Hypothetical protein |
| TP02_0914 | M | M | M | M | Hypothetical protein |
| TP02_0915 | M | M | M | M | Hypothetical protein |
| TP02_0953 | UM | UM | UM | M | Hypothetical protein |
| TP02_0960 | M | M | M | UM | Hypothetical protein |
| TP03_0923 | UM | M | UM | UM | Tpr |
| TP03_0924 | UM | M | UM | UM | Tpr |
| TP03_0925 | UM | M | UM | UM | Tpr |
| TP03_0926 | UM | M | M | UM | Tpr |
| TP03_0927 | UM | M | M | UM | Tpr |
| TP03_0928 | UM | M | M | UM | Tpr |
| TP03_0905 | UM | UM | M | UM | Tpr |
| TP03_0906 | UM | UM | M | UM | Tpr |
| TP03_0907 | UM | UM | M | UM | Tpr |
| TP03_0908 | UM | UM | M | UM | Tpr |
| TP03_0909 | UM | UM | M | UM | Tpr |
| TP03_0910 | UM | UM | M | UM | Tpr |
| TP03_0911 | UM | UM | M | UM | Tpr |
| TP03_0912 | UM | UM | M | UM | Tpr |
| TP03_0913 | UM | M | M | UM | Tpr |
| TP03_0914 | UM | M | M | UM | Tpr |
| TP03_0915 | UM | M | M | UM | Tpr |
| TP03_0916 | UM | UM | M | UM | Tpr |
| TP03_0917 | UM | UM | M | UM | Tpr |
| TP03_0918 | UM | UM | M | UM | Tpr |
| TP03_0919 | UM | UM | M | UM | Tpr |
| TP03_0920 | UM | UM | M | UM | Tpr |
| TP03_0921 | UM | UM | M | UM | Tpr |
| TP03_0922 | UM | UM | M | UM | Tpr |
| TP03_0614 | UM | M | M | UM | Tpr |
| TP03_0615 | UM | M | M | UM | Tpr |
| TP03_0616 | UM | M | M | UM | Tpr |
| TP03_0617 | UM | M | M | UM | Tpr |
| TP03_0618 | M | M | M | UM | Hypothetical protein |
| TP03_0785 | UM | UM | M | M | Hypothetical protein |
| TP03_0822 | UM | UM | UM | M | Hypothetical protein; Low complexity region |
| TP03_0871 | M | M | UM | M | Hypothetical protein |
| TP03_0872 | M | M | UM | M | Hypothetical protein |
| TP03_0875 | UM | M | UM | M | Hypothetical protein; Low complexity region |
| TP03_0876 | UM | UM | UM | M | Hypothetical protein; Low complexity region |
| TP03_0877 | UM | UM | UM | M | Hypothetical protein |
| TP03_0878 | UM | UM | M | M | Hypothetical protein |
| TP03_0879 | UM | UM | M | UM | Hypothetical protein; Low complexity region |
| TP03_0880 | M | UM | M | UM | Hypothetical protein; Low complexity region |
| TP03_0885 | M | UM | M | UM | Hypothetical protein |
| TP03_0930 | M | M | M | M | Hypothetical protein |
| TP03_0001 | M | UM | M | UM | Hypothetical protein |
| TP03_0314 | UM | M | UM | UM | Hypothetical protein |
| TP03_0338 | M | M | M | M | Hypothetical protein |
| TP03_0368 | M | M | UM | UM | Hypothetical protein |
| TP03_0478 | UM | UM | M | UM | RNA helicase |
| TP03_0480 | M | UM | M | UM | clathrin heavy chain |
| TP03_0484 | M | UM | M | UM | hypotheticalprotein; simple repeat |
| TP03_0543 | M | M | M | UM | Hypothetical protein |
| TP03_0561 | M | M | M | UM | Hypothetical protein; simple repeat |
| TP03_0562 | UM | M | M | UM | Hypothetical protein; TPRP1 |
| TP03_0563 | UM | M | M | UM | Hypothetical protein; TPRP1 |
| TP03_0564 | UM | M | M | UM | Hypothetical protein |
| TP03_0565 | M | M | M | UM | replication factor C large subunit |
| TP04_0008 | M | UM | UM | UM | Hypothetical protein |
| TP04_0009 | UM | UM | M | M | Hypothetical protein |
| TP04_0012 | UM | UM | UM | M | Hypothetical protein |
| TP04_0145 | UM | UM | UM | UM | Hypothetical protein |
| TP04_0488 | UM | UM | UM | UM | Hypothetical protein |
| TP04_0489 | UM | UM | UM | UM | Hypothetical protein |
| TP04_0490 | UM | UM | UM | UM | Hypothetical protein |
| TP04_0491 | UM | UM | UM | UM | Hypothetical protein |
| TP04_0590 | UM | UM | M | UM | Hypothetical protein |
| TP04_0923 | M | M | M | M | Hypothetical protein |
| TP04_0929 | UM | UM | UM | UM | Hypothetical protein |
| TP04_0001 | M | M | M | M | Hypothetical protein |
| TP04_0006 | UM | UM | UM | M | Hypothetical protein |
| TP04_0007 | UM | UM | UM | M | Hypothetical protein |
