# Supplementary material for: High-resolution genotyping and mapping of recombination and gene conversion in the protozoan Theileria parva using whole genome sequencing
Source: BMC Genomics. 2012 Sep 23;13:503. doi: 10.1186/1471-2164-13-503 (PMC3575351; doi:10.1186/1471-2164-13-503)
Supplement: Additional file 6: Table S1 — Crossover (CO) breakpoints and sizes of associated gene conversions (GC) in the progeny strains. [file 1471-2164-13-503-S6.doc]

## Supplementary Table 1. Crossover (CO) breakpoints and sizes of associated gene conversions (GC) in the progeny strains

| Strain | Chromosome ^1^ | Start | End | Length (bp) | Type ^2^ | GC size (bp) ^2^ | Overlapped regions ^3^ |
| --- | --- | --- | --- | --- | --- | --- | --- |
| MugugaMarikebuni | 1 | 1124549 | 1126167 | 1619 | s | 0 | TP01_0539* |
| MugugaMarikebuni | 1 | 1126172 | 1126929 | 758 | s | 0 | TP01_0539* |
| MugugaMarikebuni | 2 | 835350 | 838442 | 3093 | c | 326 | TP02_0417-TP02_0418 |
| MugugaMarikebuni | 2 | 838499 | 839259 | 761 | s | 0 | TP02_0418* |
| MugugaMarikebuni | 2 | 1573015 | 1578234 | 5220 | c | 65 | TP02_0781-TP02_0782 |
| MugugaMarikebuni | 2 | 1578234 | 1581631 | 3398 | c | 582 | TP02_0782 |
| MugugaMarikebuni | 2 | 1949617 | 1950670 | 1054 | c | 7 | TP02_0951* |
| MugugaMarikebuni | 3 | 34758 | 36287 | 1530 | c | 277 | TP03_0014-TP03_0015 |
| MugugaMarikebuni | 3 | 179370 | 182184 | 2815 | c | 975 | TP03_0093 |
| MugugaMarikebuni | 3 | 989336 | 990204 | 869 | c | 16 | TP03_0484-TP03_0485 |
| MugugaMarikebuni | 3 | 1020245 | 1021936 | 1692 | s | 0 | TP03_0483-TP03_0484 |
| MugugaMarikebuni | 3 | 1124372 | 1125811 | 1440 | s | 0 | TP03_0532* |
| MugugaMarikebuni | 3 | 1636465 | 1637081 | 617 | s | 0 | TP03_0768-TP03_0769 |
| MugugaMarikebuni | 3 | 1650287 | 1650797 | 511 | s | 0 | TP03_0780 |
| MugugaMarikebuni | 3 | 1717057 | 1720224 | 3168 | s | 0 | TP03_0817-TP03_0820 |
| MugugaUganda | 1 | 513292 | 517206 | 3915 | c | 2207 | TP01_0256-TP01_0257 |
| MugugaUganda | 1 | 517206 | 536721 | 19516 | c | 4211 | TP01_0257-TP01_0262 |
| MugugaUganda | 1 | 651512 | 656070 | 4559 | c | 19 | TP01_0318-TP01_0320 |
| MugugaUganda | 1 | 668440 | 671760 | 3321 | s | 0 | TP01_0325-TP01_0327 |
| MugugaUganda | 1 | 927928 | 929566 | 1639 | s | 0 | TP01_0456* |
| MugugaUganda | 1 | 930160 | 931626 | 1467 | c | 407 | TP01_0457-TP01_0458 |
| MugugaUganda | 1 | 1409263 | 1409972 | 710 | c | 23 | TP01_0674 |
| MugugaUganda | 1 | 1445637 | 1447624 | 1988 | s | 0 | TP01_0687* |
| MugugaUganda | 1 | 2525824 | 2537955 | 12132 | c | 10585 | TP01_1223-TP01_1227 |
| MugugaUganda | 2 | 1349 | 2599 | 1251 | c | 548 |  |
| MugugaUganda | 2 | 826517 | 830991 | 4475 | s | 0 | TP02_0413-TP02_0414 |
| MugugaUganda | 2 | 1180902 | 1182428 | 1527 | s | 0 | TP02_0581 |
| MugugaUganda | 2 | 1773727 | 1775963 | 2237 | c | 426 | TP02_0875 |
| MugugaUganda | 3 | 603988 | 611915 | 7928 | c | 3826 | TP03_0292-TP03_0295 |
| MugugaUganda | 3 | 623470 | 627839 | 4370 | c | 767 | TP03_0301-TP03_0303 |
| MugugaUganda | 3 | 639978 | 642063 | 2086 | s | 0 | TP03_0309* |
| MugugaUganda | 3 | 938570 | 943823 | 5254 | s | 0 | TP03_0897-TP03_0899 |
| MugugaUganda | 3 | 944386 | 946528 | 2143 | s | 0 | TP03_0899-TP03_0900 |
| MugugaUganda | 3 | 1157421 | 1159158 | 1738 | c | 140 | TP03_0544 |
| MugugaUganda | 3 | 1165270 | 1167110 | 1841 | c | 336 | TP03_0546-TP03_0547 |
| MugugaUganda | 3 | 1423803 | 1425788 | 1986 | c | 17 | TP03_0667 |
| MugugaUganda | 4 | 230510 | 231446 | 937 | s | 0 | TP04_0121-TP04_0122 |
| MugugaUganda | 4 | 920950 | 978901 | 57952 | s | 0 | TP04_0454-TP04_0481 |
| MugugaUganda | 4 | 1141421 | 1144961 | 3541 | s | 0 | TP04_0568-TP04_0570 |

# 1 Chromosome. For chromosome 3, the positions were calculated by concatenating contigs NC_876245, NW_876244, NW_876243, and NW_876242, without estimated gap distance. For chromosome 4, the positions were calculated by concatenating contigs NC_876247 and NW_876246, also without estimated gap distance.

# 2 “c” represents complex breakpoints with gene conversion tracks. “s” represent simple breakpoints without gene conversion. The sizes of gene conversion tracks were computed using only the markers that show allele changes.

# 3 “*” represents breakpoints inclusively fell within the listed genes. Blank cells represent tracks located in the intergenic regions between the listed genes. The rest are breakpoints that overlapped boundaries of gene and intergenic regions.
