# Supplementary material for: High-resolution genotyping and mapping of recombination and gene conversion in the protozoan Theileria parva using whole genome sequencing
Source: BMC Genomics. 2012 Sep 23;13:503. doi: 10.1186/1471-2164-13-503 (PMC3575351; doi:10.1186/1471-2164-13-503)
Supplement: Additional file 8: Table S2 — Non-crossover breakpoints in the progeny strains. [file 1471-2164-13-503-S8.doc]

**Supplementary Table 2. Non-crossover breakpoints in the progeny strains.**

| Strain | Chromosome ^1^ | Start ^3^ | End ^3^ | Length/GC size (bp) ^3^ | Regions overlapped^2^ |
| --- | --- | --- | --- | --- | --- |
| MugugaMarikebuni | 1 | 2506 | 3543 | 1038 | TP01_0001 |
| MugugaMarikebuni | 1 | 266622 | 266804 | 183 | TP01_0130* |
| MugugaMarikebuni | 1 | 642044 | 642103 | 60 | TP01_0314* |
| MugugaMarikebuni | 1 | 925186 | 926176 | 991 |  |
| MugugaMarikebuni | 1 | 1135432 | 1135542 | 111 | TP01_0544* |
| MugugaMarikebuni | 1 | 1161346 | 1161587 | 242 | TP01_0554* |
| MugugaMarikebuni | 1 | 1276778 | 1276871 | 94 |  |
| MugugaMarikebuni | 1 | 1332318 | 1332399 | 82 | TP01_0640* |
| MugugaMarikebuni | 1 | 1335148 | 1335295 | 148 | TP01_0640* |
| MugugaMarikebuni | 1 | 1409707 | 1409746 | 40 |  |
| MugugaMarikebuni | 1 | 1427616 | 1427696 | 81 |  |
| MugugaMarikebuni | 1 | 1457943 | 1458051 | 109 | TP01_0692* |
| MugugaMarikebuni | 1 | 1919076 | 1919454 | 379 |  |
| MugugaMarikebuni | 1 | 2462285 | 2462730 | 446 | TP01_1191 |
| MugugaMarikebuni | 2 | 8078 | 8119 | 42 | TP02_0003* |
| MugugaMarikebuni | 2 | 13223 | 13317 | 95 | TP02_0006* |
| MugugaMarikebuni | 2 | 15078 | 16248 | 1171 | TP02_0007* |
| MugugaMarikebuni | 2 | 38117 | 38184 | 68 |  |
| MugugaMarikebuni | 2 | 832811 | 834106 | 1296 | TP02_0415-TP02_0416 |
| MugugaMarikebuni | 2 | 838124 | 838357 | 234 | TP02_0418* |
| MugugaMarikebuni | 2 | 1052005 | 1052175 | 171 | TP02_0524* |
| MugugaMarikebuni | 2 | 1578485 | 1579397 | 913 | TP02_0782* |
| MugugaMarikebuni | 2 | 1596342 | 1596392 | 51 | TP02_0788* |
| MugugaMarikebuni | 2 | 1702683 | 1702913 | 231 | TP02_0841 |
| MugugaMarikebuni | 2 | 1778662 | 1778707 | 46 | TP02_0876* |
| MugugaMarikebuni | 2 | 1782109 | 1782153 | 45 | TP02_0876* |
| MugugaMarikebuni | 2 | 1802342 | 1802599 | 258 | TP02_0887* |
| MugugaMarikebuni | 3 | 197277 | 197319 | 43 | TP03_0102* |
| MugugaMarikebuni | 3 | 471833 | 471943 | 111 | TP03_0233* |
| MugugaMarikebuni | 3 | 653431 | 654155 | 725 | TP03_0315* |
| MugugaMarikebuni | 3 | 665748 | 665840 | 93 | TP03_0319* |
| MugugaMarikebuni | 3 | 702123 | 702131 | 9 |  |
| MugugaMarikebuni | 3 | 713264 | 716890 | 3627 | TP03_0345-TP03_0347 |
| MugugaMarikebuni | 3 | 951699 | 951780 | 82 | TP03_0463* |
| MugugaMarikebuni | 3 | 1394513 | 1395130 | 618 | TP03_0655 |
| MugugaMarikebuni | 3 | 1451356 | 1451934 | 579 | TP03_0675* |
| MugugaMarikebuni | 3 | 1702320 | 1702405 | 86 | TP03_0808* |
| MugugaMarikebuni | 3 | 1724870 | 1724914 | 45 | TP03_0822* |
| MugugaMarikebuni | 3 | 1742232 | 1742258 | 27 | TP03_0827* |
| MugugaMarikebuni | 3 | 1863748 | 1864321 | 574 | TP03_0882* |
| MugugaMarikebuni | 4 | 21491 | 21535 | 45 | TP04_0009* |
| MugugaMarikebuni | 4 | 31229 | 31731 | 503 | TP04_0014* |
| MugugaMarikebuni | 4 | 211245 | 211282 | 38 | TP04_0110* |
| MugugaMarikebuni | 4 | 278105 | 278238 | 134 |  |
| MugugaMarikebuni | 4 | 484102 | 484175 | 74 | TP04_0244* |
| MugugaMarikebuni | 4 | 813509 | 814135 | 627 | TP04_0406 |
| MugugaMarikebuni | 4 | 824185 | 824290 | 106 | TP04_0410* |
| MugugaMarikebuni | 4 | 844176 | 844251 | 76 | TP04_0420* |
| MugugaMarikebuni | 4 | 1639131 | 1639145 | 15 | TP04_0822* |
| MugugaMarikebuni | 4 | 1796848 | 1796900 | 53 | TP04_0905* |
| MugugaMarikebuni | 4 | 1851491 | 1851728 | 238 |  |
| MugugaUganda | 1 | 223983 | 228083 | 4101 | TP01_0110-TP01_0111 |
| MugugaUganda | 1 | 560799 | 561436 | 638 | TP01_0273 |
| MugugaUganda | 1 | 943506 | 946593 | 3088 | TP01_0462-TP01_0463 |
| MugugaUganda | 1 | 1093554 | 1093929 | 376 | TP01_0526* |
| MugugaUganda | 1 | 1097259 | 1097621 | 363 | TP01_0527 |
| MugugaUganda | 1 | 1103308 | 1103362 | 55 | TP01_0530* |
| MugugaUganda | 1 | 1134809 | 1134995 | 187 | TP01_0544* |
| MugugaUganda | 1 | 1161346 | 1161587 | 242 | TP01_0554* |
| MugugaUganda | 1 | 1193660 | 1193954 | 295 |  |
| MugugaUganda | 1 | 1285241 | 1285301 | 61 |  |
| MugugaUganda | 1 | 1311679 | 1311751 | 73 | TP01_0627* |
| MugugaUganda | 1 | 1405373 | 1405427 | 55 | TP01_0670* |
| MugugaUganda | 1 | 1454122 | 1454168 | 47 | TP01_0690* |
| MugugaUganda | 1 | 2474097 | 2474257 | 161 |  |
| MugugaUganda | 1 | 2534479 | 2535061 | 583 | TP01_1227* |
| MugugaUganda | 1 | 2536679 | 2537520 | 842 | TP01_1227 |
| MugugaUganda | 2 | 2570 | 2586 | 17 |  |
| MugugaUganda | 2 | 13481 | 13609 | 129 | TP02_0006* |
| MugugaUganda | 2 | 34029 | 34473 | 445 | TP02_0016* |
| MugugaUganda | 2 | 38117 | 38191 | 75 |  |
| MugugaUganda | 2 | 92969 | 95014 | 2046 | TP02_0038* |
| MugugaUganda | 2 | 549923 | 549954 | 32 | TP02_0274* |
| MugugaUganda | 2 | 720900 | 721001 | 102 | TP02_0363* |
| MugugaUganda | 2 | 837775 | 837798 | 24 | TP02_0418* |
| MugugaUganda | 2 | 1384716 | 1385504 | 789 | TP02_0691* |
| MugugaUganda | 2 | 1577575 | 1577728 | 154 | TP02_0782* |
| MugugaUganda | 2 | 1579971 | 1581535 | 1565 | TP02_0782 |
| MugugaUganda | 2 | 1593742 | 1595433 | 1692 | TP02_0787-TP02_0788 |
| MugugaUganda | 2 | 1632988 | 1633108 | 121 | TP02_0802* |
| MugugaUganda | 2 | 1633707 | 1633723 | 17 | TP02_0802* |
| MugugaUganda | 2 | 1635397 | 1635427 | 31 | TP02_0802* |
| MugugaUganda | 3 | 224646 | 225141 | 496 | TP03_0114* |
| MugugaUganda | 3 | 635939 | 636905 | 967 | TP03_0306-TP03_0307 |
| MugugaUganda | 3 | 697030 | 697685 | 656 | TP03_0338* |
| MugugaUganda | 3 | 707212 | 707346 | 135 | TP03_0342* |
| MugugaUganda | 3 | 743749 | 757795 | 14047 | TP03_0360-TP03_0368 |
| MugugaUganda | 3 | 951796 | 951857 | 62 | TP03_0463* |
| MugugaUganda | 3 | 1019504 | 1019790 | 287 | TP03_0483* |
| MugugaUganda | 3 | 1130626 | 1130636 | 11 | TP03_0534* |
| MugugaUganda | 3 | 1156241 | 1156563 | 323 | TP03_0543* |
| MugugaUganda | 3 | 1201857 | 1201874 | 18 | TP03_0560* |
| MugugaUganda | 3 | 1416284 | 1417607 | 1324 | TP03_0663-TP03_0664 |
| MugugaUganda | 3 | 1625369 | 1631916 | 6548 |  |
| MugugaUganda | 3 | 1843051 | 1851501 | 8451 | TP03_0871-TP03_0875 |
| MugugaUganda | 3 | 1870740 | 1870947 | 208 | TP03_0885 |
| MugugaUganda | 4 | 279301 | 279918 | 618 | TP04_0145* |
| MugugaUganda | 4 | 469017 | 469062 | 46 |  |
| MugugaUganda | 4 | 484102 | 484175 | 74 | TP04_0244* |
| MugugaUganda | 4 | 826601 | 826761 | 161 | TP04_0412* |
| MugugaUganda | 4 | 829336 | 831658 | 2323 | TP04_0414 |
| MugugaUganda | 4 | 1639131 | 1639149 | 19 | TP04_0822* |
| MugugaUganda | 4 | 1641152 | 1641379 | 228 |  |
| MugugaUganda | 4 | 1669389 | 1669421 | 33 | TP04_0837* |
| MugugaUganda | 4 | 1843760 | 1844072 | 313 | TP04_0929* |
| MugugaUganda | 4 | 1847697 | 1847883 | 187 | TP04_0929* |

# 1 Chromosome. For chromosome 3, the positions were calculated by concatenating contigs NC_876245, NW_876244, NW_876243, and NW_876242, without estimated gap distance. For chromosome 4, the positions were calculated by concatenating contigs NC_876247 and NW_876246, also without estimated gap distance.

# 2 “*” represents breakpoints inclusively fell within the listed genes. Blank cells represent tracks located in the intergenic regions between the listed genes. The rest are breakpoints that overlapped boundaries of genes and intergenic regions.

3. The start, end, and size of a gene conversion track were computed using only the markers that showed allele changes (Supplementary Figure S7 C).
