# Supplementary material for: High-resolution genotyping and mapping of recombination and gene conversion in the protozoan Theileria parva using whole genome sequencing
Source: BMC Genomics. 2012 Sep 23;13:503. doi: 10.1186/1471-2164-13-503 (PMC3575351; doi:10.1186/1471-2164-13-503)
Supplement: Additional file 10: Figure S3 — Distribution of Ka/Ks ratios by intra-species polymorphism analysis. Red bars represent histograms of Ka/Ks ratios. Blue curves are normal distribution curves fit to the corresponding histograms. The top four graphs were based on BLAT alignment of Muguga mRNAs to de novo assemblies, bottom four were to mapped assemblies. [file 1471-2164-13-503-S10.pdf]

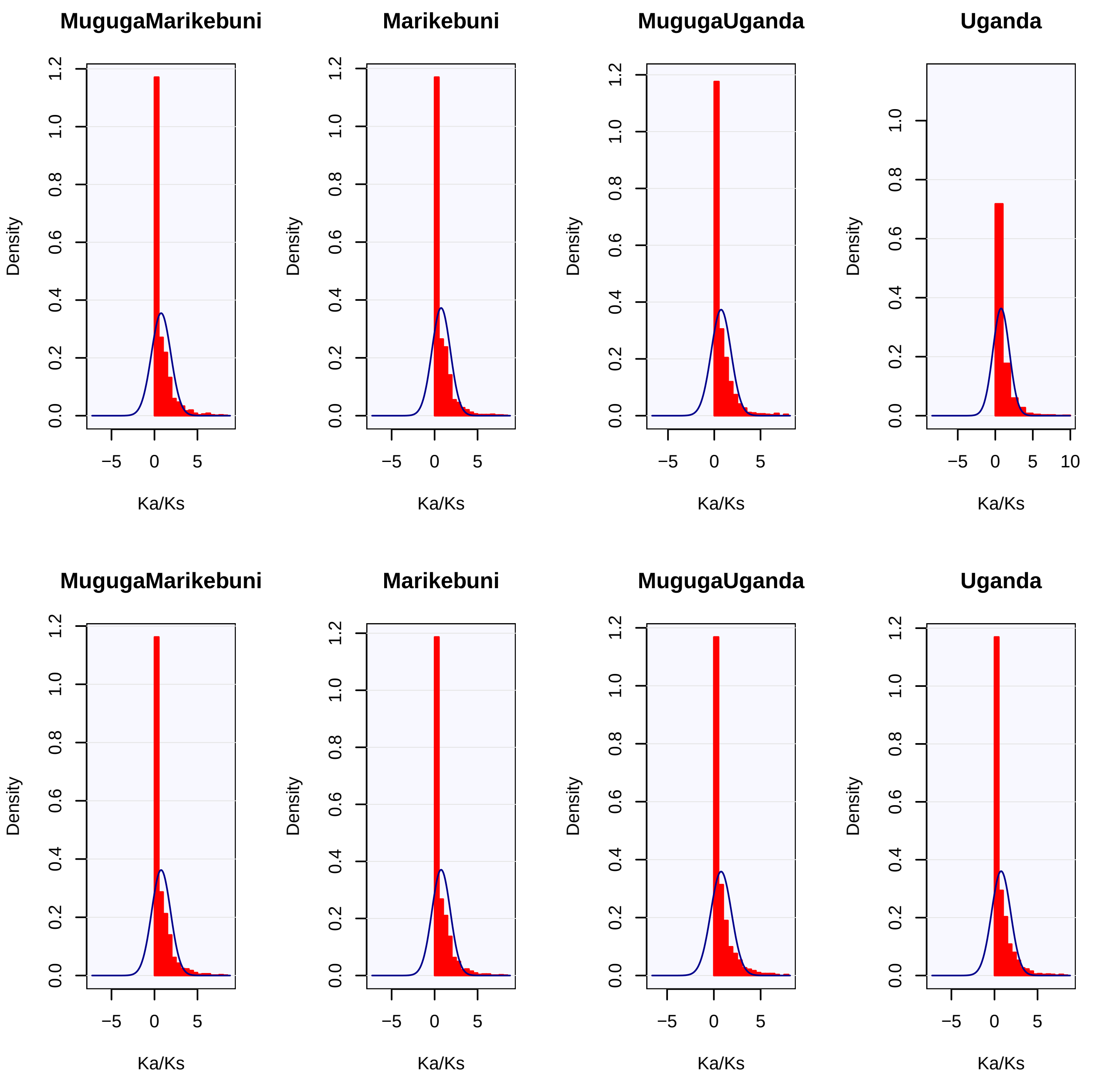
MugugaMarikebuni
Marikebuni
MugugaUganda
Uganda
Density
Ka/Ks
0.0
0.2
0.4
0.6
0.8
1.0
1.2
−5
0
5
10
MugugaMarikebuni
Marikebuni
MugugaUganda
Uganda
