# Supplementary material for: High-resolution genotyping and mapping of recombination and gene conversion in the protozoan Theileria parva using whole genome sequencing
Source: BMC Genomics. 2012 Sep 23;13:503. doi: 10.1186/1471-2164-13-503 (PMC3575351; doi:10.1186/1471-2164-13-503)
Supplement: Additional file 11: Figure S4 — Ka/Ks ratios by Fisher exact test P-values. Vertical lines represent the means of Ka/Ks ratios. Horizontal lines represent the cut-off P-value (0.05). The top four graphs were based on BLAT alignment of Muguga mRNAs to de novo assemblies, bottom four were to mapped assemblies. [file 1471-2164-13-503-S11.pdf]

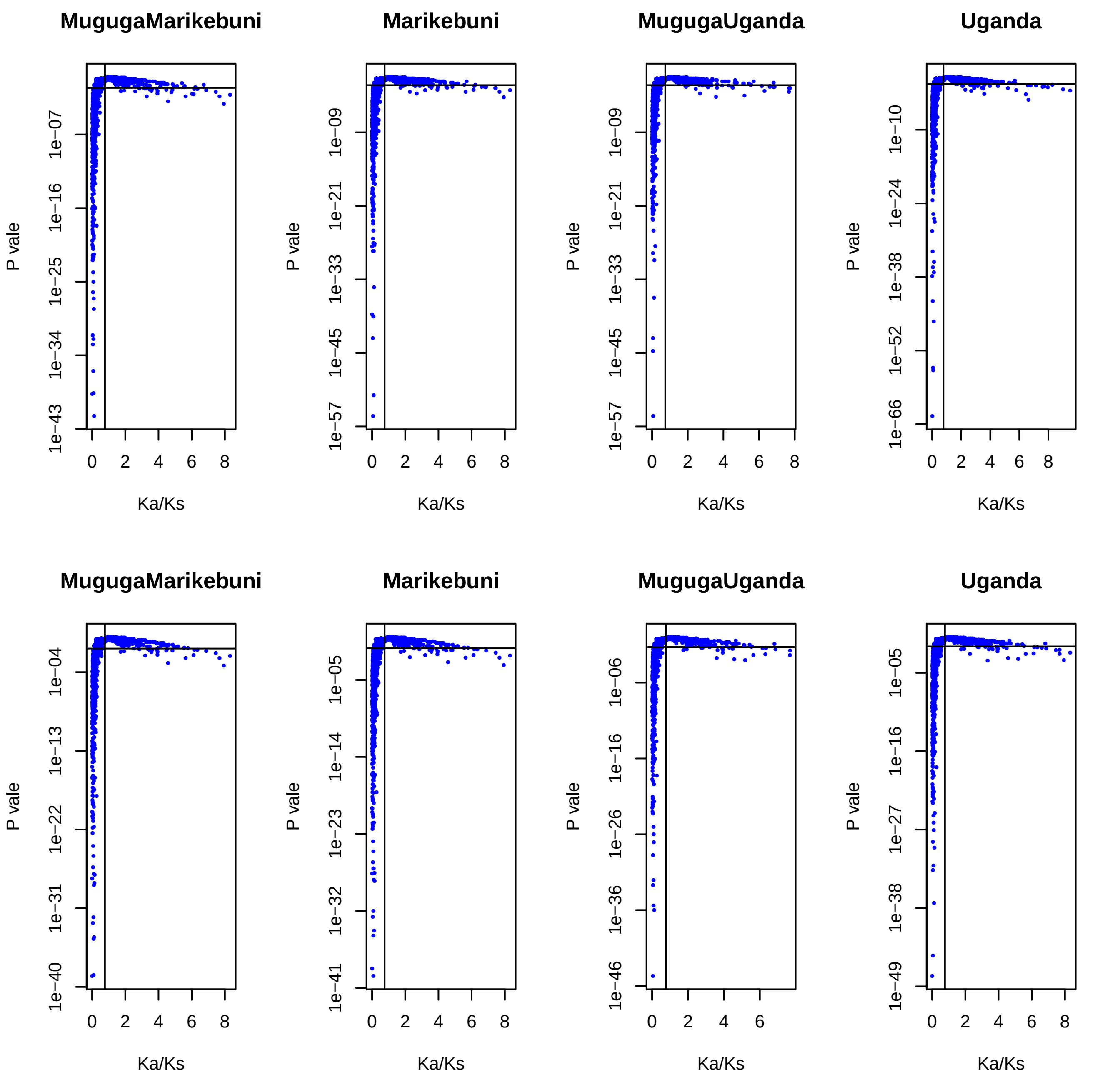
MugugaMarikebuni
Marikebuni
MugugaUganda
Uganda
P vale
Ka/Ks
1e−07
1e−16
1e−25
1e−34
1e−43
1e−09
1e−21
1e−33
1e−45
1e−57
1e−10
1e−24
1e−38
1e−52
1e−66
0
2
4
6
8
MugugaMarikebuni
Marikebuni
MugugaUganda
Uganda
1e−04
1e−13
1e−22
1e−31
1e−40
1e−05
1e−14
1e−23
1e−32
1e−41
1e−06
1e−16
1e−26
1e−36
1e−46
1e−05
1e−16
1e−27
1e−38
1e−49
