# Supplementary material for: High-resolution genotyping and mapping of recombination and gene conversion in the protozoan Theileria parva using whole genome sequencing
Source: BMC Genomics. 2012 Sep 23;13:503. doi: 10.1186/1471-2164-13-503 (PMC3575351; doi:10.1186/1471-2164-13-503)
Supplement: Additional file 12: Table S4 — Genes potentially under positive selection by intra-species polymorphism analysis. [file 1471-2164-13-503-S12.doc]

**Supplementary Table 4. Genes potentially under positive selection by intra-species polymorphism analysis**

| Genes | Protein Product | Ka/Ks  Marikebuni | Ka/Ks  MugugaMarikebuni | Ka/Ks  MugugaUganda | Ka/Ks  Uganda | KOG | KOG description | E-value | Functional Category |
| --- | --- | --- | --- | --- | --- | --- | --- | --- | --- |
| TP01_0241 | chaperonin 60 kDa | 7.68 | 7.68 | 7.68 | 7.68 | KOG0358 | Chaperonin complex component, TCP-1 delta subunit (CCT4) [Posttranslational modification, protein turnover, chaperones]. | 0 | [O] |
| TP01_0299 | hypothetical protein | 6.12 | 6.12 | 7.68 | 7.68 | KOG1489 | Predicted GTP-binding protein (ODN superfamily) [General function prediction only]. | 4.00E-27 | [R] |
| TP01_0544 | RNA helicase |  |  | 3.93 | 3.93 | KOG0922 | DEAH-box RNA helicase [RNA processing and modification]. | 0 | [A] |
| TP01_0558 | hypothetical protein |  |  | 6.61 | 6.61 | KOG2056 | Equilibrative nucleoside transporter protein [Nucleotide transport and metabolism]. | 5.00E-14 | [F] |
| TP01_0686 | 60S ribosomal protein L15 |  |  |  | 7.15 | KOG2648 | Diphthamide biosynthesis protein [Translation, ribosomal structure and biogenesis]. | 2.00E-20 | [J] |
| TP01_0709 | 26S proteasome regulatory subunit 2 |  |  |  | 2.10 | KOG2005 | 26S proteasome regulatory complex, subunit RPN1/PSMD2 [Posttranslational modification, protein turnover, chaperones]. | 8.00E-75 | [O] |
| TP01_0720 | P-type ATPase |  |  |  | 9.02 | KOG0202 | Ca2+ transporting ATPase [Inorganic ion transport and metabolism]. | 2.00E-81 | [P] |
| TP01_0794 | vacuolar sorting protein 35 | 3.63 | 3.63 |  |  | KOG1107 | Membrane coat complex Retromer, subunit VPS35 [Intracellular trafficking, secretion, and vesicular transport]. | 5.00E-107 | [U] |
| TP01_0797 | hypothetical protein |  |  |  | 3.24 | KOG0495 | HAT repeat protein [RNA processing and modification]. | 0 | [A] |
| TP01_0945 | polyadenylate binding protein | 4.48 | 4.48 | 4.49 | 4.49 | KOG0123 | Polyadenylate-binding protein (RRM superfamily) [RNA processing and modification, Translation, ribosomal structure and biogenesis]. | 4.00E-99 | [A] [J] |
| TP01_0961 | hypothetical protein | 3.47 | 3.47 | 6.31 | 6.11 | KOG0927 | Predicted transporter (ABC superfamily) [General function prediction only]. | 4.00E-14 | [R] |
| TP01_1019 | ATP-dependent RNA helicase |  |  | 2.82 | 2.82 | KOG0925 | mRNA splicing factor ATP-dependent RNA helicase [RNA processing and modification]. | 0 | [A] |
| TP01_1050 | aconitatehydratase | 3.51 | 3.51 | 5.19 | 5.19 | KOG0452 | RNA-binding translational regulator IRP (aconitase superfamily) [RNA processing and modification, Translation, ribosomal structure and biogenesis]. | 0 | [A] [J] |
| TP01_1107 | myosin B |  |  |  | 3.42 | KOG0161 | Myosin class II heavy chain [Cytoskeleton]. | 6.00E-130 | [Z] |
| TP01_1121 | hypothetical protein |  |  |  | 2.94 | KOG2280 | Vacuolar assembly/sorting protein VPS16 [Intracellular trafficking, secretion, and vesicular transport]. | 6.00E-21 | [U] |
| TP01_1132 | ATP-dependent helicase |  |  |  | 2.12 | KOG0391 | SNF2 family DNA-dependent ATPase [General function prediction only]. | 1.00E-120 | [R] |
| TP02_0146 | hypothetical protein | 6.88 | 6.88 | 6.88 | 6.88 | KOG2535 | RNA polymerase II elongator complex, subunit ELP3/histone acetyltransferase [Chromatin structure and dynamics, Transcription]. | 2.00E-80 | [B] [K] |
| TP02_0337 | hypothetical protein |  |  | 4.30 | 4.30 | KOG2048 | WD40 repeat protein [General function prediction only]. | 5.00E-34 | [R] |
| TP02_0382 | hypothetical protein |  |  |  | 2.09 | KOG0973 | Histone transcription regulator HIRA, WD repeat superfamily [Cell cycle control, cell division, chromosome partitioning, Transcription]. | 6.00E-22 | [D] [K] |
| TP02_0462 | hypothetical protein |  |  |  | 5.80 | KOG0581 | Mitogen-activated protein kinase kinase (MAP2K) [Signal transduction mechanisms]. | 2.00E-06 | [T] |
| TP02_0500 | phosphate transporter | 7.45 | 7.45 |  | 7.45 | KOG2493 | Na+/Pi symporter [Inorganic ion transport and metabolism]. | 4.00E-41 | [P] |
| TP02_0577 | 6-phosphofructokinase |  |  |  | 6.63 | KOG2440 | Pyrophosphate-dependent phosphofructo-1-kinase [Carbohydrate transport and metabolism]. | 5.00E-49 | [G] |
| TP02_0613 | ATP-dependent RNA helicase | 6.85 |  |  |  | KOG0326 | ATP-dependent RNA helicase [RNA processing and modification]. | 0 | [A] |
| TP02_0749 | hypothetical protein |  |  | 3.59 | 3.59 | KOG1896 | mRNA cleavage and polyadenylation factor II complex, subunit CFT1 (CPSF subunit) [RNA processing and modification]. | 5.00E-06 | [A] |
| TP02_0771 | DNA gyrase subunit A | 5.64 | 5.64 | 5.64 | 5.64 | KOG0355 | DNA topoisomerase type II [Chromatin structure and dynamics]. | 3.00E-15 | [B] |
| TP02_0809 | hypothetical protein |  |  | 2.73 | 2.73 | KOG2156 | Tubulin-tyrosine ligase-related protein [Posttranslational modification, protein turnover, chaperones]. | 3.00E-31 | [O] |
| TP02_0885 | hypothetical protein | 7.94 | 7.94 |  | 7.94 | KOG3869 | Uncharacterized conserved protein [Function unknown]. | 2.00E-06 | [S] |
| TP03_0108 | syntaxin binding protein | 2.54 | 2.54 |  |  | KOG1300 | Vesicle trafficking protein Sec1 [Intracellular trafficking, secretion, and vesicular transport]. | 8.00E-37 | [U] |
| TP03_0165 | ABC transporter |  | 3.13 |  | 7.97 | KOG0054 | Multidrug resistance-associated protein/mitoxantrone resistance protein, ABC superfamily [Secondary metabolites biosynthesis, transport and catabolism]. | 1.00E-58 | [Q] |
| TP03_0369 | hypothetical protein | 3.20 | 3.20 |  |  | KOG3660 | Sodium-neurotransmitter symporter [Signal transduction mechanisms]. | 9.00E-24 | [T] |
| TP03_0375 | integral membrane protein |  |  |  | 8.28 | KOG0209 | P-type ATPase [Inorganic ion transport and metabolism]. | 5.00E-104 | [P] |
| TP03_0480 | clathrin heavy chain | 1.74 |  |  |  | KOG0985 | Vesicle coat protein clathrin, heavy chain [Intracellular trafficking, secretion, and vesicular transport]. | 0 | [U] |
| TP03_0491 | DNA mismatch repair protein PMS1 |  | 3.29 |  | 2.90 | KOG1978 | DNA mismatch repair protein - MLH2/PMS1/Pms2 family [Replication, recombination and repair]. | 4.00E-63 | [L] |
| TP03_0511 | cGMP-dependent protein kinase |  |  | 3.21 | 3.21 | KOG0614 | cGMP-dependent protein kinase [Signal transduction mechanisms]. | 2.00E-98 | [T] |
| TP03_0532 | ATP-dependent RNA helicase |  | 2.60 |  |  | KOG0334 | RNA helicase [RNA processing and modification]. | 6.00E-141 | [A] |
| TP03_0534 | hypothetical protein |  |  |  | 3.52 | KOG1427 | Uncharacterized conserved protein, contains RCC1 domain [Function unknown]. | 3.00E-22 | [S] |
| TP03_0539 | NADH dehydrogenase |  |  |  | 7.61 | KOG2495 | NADH-dehydrogenase (ubiquinone) [Energy production and conversion]. | 1.00E-80 | [C] |
| TP03_0549 | DNA ligase I |  |  |  | 6.45 | KOG0967 | ATP-dependent DNA ligase I [Replication, recombination and repair]. | 0 | [L] |
| TP03_0575 | hypothetical protein | 4.76 |  |  |  | KOG1625 | DNA polymerase alpha-primase complex, polymerase-associated subunit B [Replication, recombination and repair]. | 6.00E-10 | [L] |
| TP03_0649 | hypothetical protein |  |  | 2.45 |  | KOG4674 | Uncharacterized conserved coiled-coil protein [Function unknown]. | 5.00E-10 | [S] |
| TP03_0751 | hypothetical protein | 2.28 |  |  | 2.28 | KOG0954 | PHD finger protein [General function prediction only]. | 9.00E-07 | [R] |
| TP03_0808 | hypothetical protein | 3.97 |  |  | 3.97 | KOG2602 | Predicted cell surface protein homologous to bacterial outer membrane proteins [General function prediction only]. | 7.00E-26 | [R] |
| TP03_0826 | hypothetical protein |  | 3.35 |  |  | KOG0323 | TFIIF-interacting CTD phosphatases, including NLI-interacting factor [Transcription]. | 9.00E-15 | [K] |
| TP03_0830 | ATP-dependent RNA helicase | 2.60 |  |  | 2.61 | KOG0331 | ATP-dependent RNA helicase [RNA processing and modification]. | 8.00E-118 | [A] |
| TP04_0057 | DNA replication licensing factor MCM4 | 3.59 |  |  | 3.59 | KOG0478 | DNA replication licensing factor, MCM4 component [Replication, recombination and repair]. | 2.00E-118 | [L] |
| TP04_0363 | hypothetical protein | 7.43 |  | 7.74 | 7.74 | KOG1410 | Nuclear transport receptor RanBP16 (importin beta superfamily) [Nuclear structure, Intracellular trafficking, secretion, and vesicular transport]. | 3.00E-157 | [Y] [U] |
| TP04_0573 | hypothetical protein | 4.58 | 4.58 | 4.58 | 4.58 | KOG0955 | PHD finger protein BR140/LIN-49 [General function prediction only]. | 8.00E-13 | [R] |
| TP04_0665 | hypothetical protein | 1.74 | 1.74 | 1.74 | 1.74 | KOG0889 | Histone acetyltransferase SAGA, TRRAP/TRA1 component, PI-3 kinase superfamily [Signal transduction mechanisms, Chromatin structure and dynamics, Replication, recombination and repair, Cell cycle control, cell division, chromosome partitioning]. | 9.00E-42 | [T] [B] [L] [D] |
| TP04_0667 | hypothetical protein | 6.34 | 6.34 | 6.34 | 6.34 | KOG2191 | RNA-binding protein NOVA1/PASILLA and related KH domain proteins [RNA processing and modification, General function prediction only]. | 5.00E-07 | [A] [R] |
| TP04_0806 | ubiquinone/menaquinone biosynthesis methyltransferase | 6.35 | 6.35 | 6.35 | 6.35 | KOG1540 | Ubiquinone biosynthesis methyltransferase COQ5 [Coenzyme transport and metabolism]. | 4.00E-59 | [H] |
| TP04_0813 | DNA polymerase epsilon, catalytic subunit A | 1.87 |  | 1.87 |  | KOG1798 | DNA polymerase epsilon, catalytic subunit A [Replication, recombination and repair]. | 2.00E-146 | [L] |
| TP04_0848 | hypothetical protein | 6.79 |  | 6.79 | 6.79 | KOG2268 | Serine/threonine protein kinase [Signal transduction mechanisms, General function prediction only]. | 3.00E-108 | [T] [R] |
| TP04_0875 | hypothetical protein | 2.70 |  | 2.70 | 2.70 | KOG0928 | Pattern-formation protein/guanine nucleotide exchange factor [Intracellular trafficking, secretion, and vesicular transport]. | 4.00E-32 | [U] |
| TP04_0906 | hypothetical protein | 3.95 | 3.95 | 3.95 | 3.95 | KOG0128 | RNA-binding protein SART3 (RRM superfamily) [RNA processing and modification]. | 8.00E-24 | [A] |
| TP04_0908 | importin alpha | 3.88 | 3.88 | 3.88 | 3.88 | KOG0166 | Karyopherin (importin) alpha [Intracellular trafficking, secretion, and vesicular transport]. | 9.00E-154 | [U] |
| TP01_0022 | hypothetical protein | 3.58 | 3.58 |  |  |  |  |  |  |
| TP01_0257 | hypothetical protein |  |  | 3.60 | 3.35 |  |  |  |  |
| TP01_0263 | hypothetical protein | 1.73 | 1.73 | 1.90 | 1.90 |  |  |  |  |
| TP01_0428 | hypothetical protein | 2.85 | 2.85 |  |  |  |  |  |  |
| TP01_0431 | hypothetical protein | 4.84 | 4.84 |  |  |  |  |  |  |
| TP01_0671 | hypothetical protein |  |  | 3.65 | 3.65 |  |  |  |  |
| TP01_0741 | hypothetical protein |  |  |  | 2.97 |  |  |  |  |
| TP01_0795 | hypothetical protein |  |  |  | 9.50 |  |  |  |  |
| TP01_1001 | hypothetical protein |  |  |  | 3.16 |  |  |  |  |
| TP01_1126 | hypothetical protein | 6.16 | 6.16 | 6.16 | 6.16 |  |  |  |  |
| TP01_1185 | hypothetical protein |  |  |  | 2.79 |  |  |  |  |
| TP02_0174 | sporozoite surface molecule protein | 4.32 | 4.53 | 3.34 | 3.59 |  |  |  |  |
| TP02_0569 | hypothetical protein |  |  |  | 3.03 |  |  |  |  |
| TP02_0762 | hypothetical protein | 3.94 | 3.94 | 3.94 | 3.94 |  |  |  |  |
| TP02_0782 | hypothetical protein |  |  | 3.14 | 3.14 |  |  |  |  |
| TP02_0931 | hypothetical protein | 8.32 | 8.32 |  | 8.32 |  |  |  |  |
| TP03_0019 | hypothetical protein | 2.88 |  |  |  |  |  |  |  |
| TP03_0143 | hypothetical protein |  | 4.78 |  |  |  |  |  |  |
| TP03_0149 | hypothetical protein |  | 6.04 |  |  |  |  |  |  |
| TP03_0153 | hypothetical protein |  | 3.17 |  | 5.22 |  |  |  |  |
| TP03_0242 | hypothetical protein |  |  |  | 3.42 |  |  |  |  |
| TP03_0392 | hypothetical protein |  |  |  | 3.20 |  |  |  |  |
| TP04_0120 | hypothetical protein |  | 3.25 |  |  |  |  |  |  |
| TP04_0140 | hypothetical protein | 1.93 | 1.93 | 1.93 | 1.93 |  |  |  |  |
| TP04_0435 | hypothetical protein | 4.53 |  | 4.53 | 4.53 |  |  |  |  |
| TP04_0574 | hypothetical protein | 6.60 |  | 6.60 | 6.60 |  |  |  |  |
