# Supplementary material for: High-resolution genotyping and mapping of recombination and gene conversion in the protozoan Theileria parva using whole genome sequencing
Source: BMC Genomics. 2012 Sep 23;13:503. doi: 10.1186/1471-2164-13-503 (PMC3575351; doi:10.1186/1471-2164-13-503)
Supplement: Additional file 13 — Supplementary information on Material and Methods. [file 1471-2164-13-503-S13.doc]

**Supplementary information on Material and Methods**

Cattle: Fourteen Boran calves negative to *T. parva* antibodies were used in the experiments described. Ten were infected as described and four retained as sources of lymphocytes for *in vitro* infections.

Parasites: The following stabilates were used:

*T. parva* Muguga (3087), *T.parva* Uganda (3066), *T. parva* Marikebuni (3014).

Ticks: *R. appendiculatus* (Muguga stock).

Experimental procedures:

# Muguga-Uganda co-infection

Two calves, G136 and G138 were inoculated with both *T. parva* Muguga (3087), at a stabilate dilution of 1:100 and *T. parva* Uganda (3066) undiluted. Calf G139 was inoculated only with *T. parva* Muguga (3087), at a stabilate dilution of 1:100.Calf G141 was inoculated only with *T. parva* Uganda (3066) undiluted. The Muguga stock was always inoculated over the right parotid lymph gland and the Uganda stock over the left parotid lymph gland.

# Muguga-Marikebuni co-infection

Two calves, BH284 and BH293 were inoculated with both *T. parva* Muguga (3087), at a stabilate dilution of 1:50 and *T. parva* Marikebuni (3014) undiluted. Calves BH330 and BH332 was inoculated only with *T. parva* Muguga (3087), at a stabilate dilution of 1:50.Calves BH313 and BH315 were inoculated only with *T. parva* Marikebuni (3014) undiluted. The Muguga stock was always inoculated over the left parotid lymph gland and the Marikebuni stock over the right parotid lymph gland.

Experimental Monitoring

Cattle:

1. Pre-infection blood samples for serum and then weekly samples.
2. Daily rectal temperature
3. Smears from both parotid lymph glands from day 5 for staining with Giemsa and examination for presence of schizonts.
4. Smears from both pre-scapular lymph glands from the first detection of schizonts in the pre-scapular glands.
5. Nymphal tick application from day 8 and additionally every 3^rd^ day to cover feeding during the period of piroplasms parasitaemia.
6. Lymph node biopsies for tissue culture and PCR from the local drainage lymph node during early parasitosis.
7. Blood samples for PCR analysis on alternate days once the local drainage lymph node has schizonts.

Ticks:

1. Engorged nymphal ticks were collected daily and maintained until they had moulted and hardened.
2. Tick batches were dissected, salivary glands stained and selected for those batches showing a high frequency of singly infected acinar cells.
3. Ticks from these batches were dissected and glands with single infected acini were selected using interference contrast microscopy.

Bovine lymphocyte infection for culture:

1. A salivary gland containing a single infected was crushed using the rubber base of a plunger from a 2ml syringe, with 500ul of L15 culture growth medium.
2. This medium was collected and used to infect pellets of bovine lymphocytes, either as undiluted (300ul for 5 x 10^6^ lymphocytes) or diluted 1:10 (100ul for 2 x 10^6^ lymphocytes) similar medium. This dilution series was continued down to 10^-5^ and the series from undiluted to 10^-5^ for PCR studies.

PCR /DNA studies:

Experimental approach:

The following experimental approach was used:

1. Infections, with each *T. parva* stock, were assessed in cattle with PCR and stock-specific oligonucleotide hybridisation.
2. Pick up of infections in ticks was only attempted when an animal was demonstrated to be infected with both inoculated stocks.
3. Following tick dropping and moulting tick batches were examined for infections in salivary gland acini. Batches with low infection rates were chosen to ensure that the infected acinus selected was infected with sporozoites derived from one kinete.
4. An individual acinus was used to establish infection in lymphocytes. Several infected acini were used from each tick batch (i.e. mixed infections and the two individual infections as controls).
5. Selection of material for processing was based on the use of the two oligonucleotide probes specific for each of the two stocks used in the cattle infection. This procedure did not distinguish recombinants from mixtures resulting from tick infection with kinetes from each stock, but would eliminate infections that are neither mixed nor recombined.
6. Bovine lymphocytes were infected with serial dilutions of sporozoites, so that they could be used suitable for autologous infections.
7. From mixed infections of cattle, only cell lines demonstrating both inoculated *T. parva* genomes were processed, with others lines cryopreserved.
8. Cell lines demonstrating the presence of *T. parva* recombinants were bulked for autologous infections of cattle.
